# Antarctica and the strategic plan for biodiversity

**DOI:** 10.1371/journal.pbio.2001656

**Published:** 2017-03-28

**Authors:** Steven L. Chown, Cassandra M. Brooks, Aleks Terauds, Céline Le Bohec, Céline van Klaveren-Impagliazzo, Jason D. Whittington, Stuart H. M. Butchart, Bernard W. T. Coetzee, Ben Collen, Peter Convey, Kevin J. Gaston, Neil Gilbert, Mike Gill, Robert Höft, Sam Johnston, Mahlon C. Kennicutt, Hannah J. Kriesell, Yvon Le Maho, Heather J. Lynch, Maria Palomares, Roser Puig-Marcó, Peter Stoett, Melodie A. McGeoch

**Affiliations:** 1 School of Biological Sciences, Monash University, Melbourne, Victoria, Australia; 2 School of Earth, Energy and Environmental Sciences, Stanford University, Stanford, California, United States of America; 3 Australian Antarctic Division, Department of the Environment and Energy, Kingston, Tasmania, Australia; 4 Centre Scientifique de Monaco (CSM), Département de Biologie Polaire, Monaco, Principality of Monaco; 5 Université de Strasbourg (UdS), Centre National de la Recherche Scientifique (CNRS), Institut Pluridisciplinaire Hubert Curien (IPHC) UMR 7178, Strasbourg, France; 6 Laboratoire International Associé LIA 647 *BioSensib* (CSM-CNRS-UdS), Monaco, Principality of Monaco; 7 Direction des Affaires Internationales, Département des Relations Extérieures et de la Coopération, Ministère d’État, Gouvernement Princier, Monaco, Principality of Monaco; 8 Centre for Ecological and Evolutionary Synthesis, Department of Biosciences, University of Oslo, Oslo, Norway; 9 BirdLife International, Cambridge, United Kingdom; 10 Department of Zoology, University of Cambridge, Cambridge, United Kingdom; 11 Centre for Biodiversity & Environment Research, Department of Genetics, Evolution & Environment, University College London, London, United Kingdom; 12 British Antarctic Survey, NERC, Cambridge, United Kingdom; 13 Environment & Sustainability Institute, University of Exeter, Penryn, United Kingdom; 14 Antarctica New Zealand, Christchurch, New Zealand; 15 Polar Knowledge Canada, Government of Canada, Ottawa, Ontario, Canada; 16 Secretariat of the Convention on Biological Diversity, Montreal, Québec, Canada; 17 Melbourne Law School, The University of Melbourne, Melbourne, Victoria, Australia; 18 Texas A&M University, College Station, Texas, United States of America; 19 Department of Ecology & Evolution, Stony Brook University, Stony Brook, New York, United States of America; 20 Sea Around Us, Global Fisheries Cluster, Institute for the Oceans and Fisheries, University of British Columbia, Vancouver, British Columbia, Canada; 21 Faculty of Law, University of Barcelona, Barcelona, Spain; 22 Loyola Sustainability Research Centre, Concordia University, Montreal, Québec, Canada

## Abstract

The Strategic Plan for Biodiversity, adopted under the auspices of the Convention on Biological Diversity, provides the basis for taking effective action to curb biodiversity loss across the planet by 2020—an urgent imperative. Yet, Antarctica and the Southern Ocean, which encompass 10% of the planet’s surface, are excluded from assessments of progress against the Strategic Plan. The situation is a lost opportunity for biodiversity conservation globally. We provide such an assessment. Our evidence suggests, surprisingly, that for a region so remote and apparently pristine as the Antarctic, the biodiversity outlook is similar to that for the rest of the planet. Promisingly, however, much scope for remedial action exists.

## Introduction

The Convention on Biological Diversity (CBD) is an international agreement established to sustain the diversity of life on Earth. In 2010, following assessments showing that over the previous decade the state of global biodiversity continued to decline [1], the Strategic Plan for Biodiversity 2011–2020 [2] (hereafter the Strategic Plan) was developed under the aegis of the CBD. The specific aim of the Strategic Plan is to take effective and urgent action to halt biodiversity loss, to ensure that by 2020 ecosystems are resilient and continue to provide essential services, thus securing the variety of Earth’s life and contributing to human well-being and poverty eradication. Its stated intent is to provide an overarching framework for the assessment and protection of biodiversity, not only for the entire United Nations system but for all partners engaged in biodiversity management and policy development [2].

Five Strategic Goals form the foundation of the Strategic Plan: (A) address the underlying causes of biodiversity loss by mainstreaming biodiversity across government and society; (B) reduce the direct pressures on biodiversity and promote sustainable use; (C) improve the status of biodiversity by safeguarding ecosystems, species, and genetic diversity; (D) enhance the benefits to all from biodiversity and ecosystem services; and (E) enhance implementation through participatory planning, knowledge management, and capacity building. Twenty targets, distributed across these five goals and known as the Aichi Biodiversity Targets [2] (hereafter Aichi targets), were developed for 2020. Their purpose is to help realise the Strategic Plan’s global aims and vision and to offer a flexible framework for addressing national needs and priorities. Together, the Strategic Goals and the Aichi targets both form the basis for national and regional implementation actions and provide means for assessing progress towards a halt in biodiversity loss by 2050 [3].

Aichi target-based assessments of the state of global biodiversity [3,4] pay limited attention to Antarctica and the Southern Ocean (principally south of the Antarctic Polar Front). Thus, despite its large area (~50 million km^2^; ca. 10% of the planet’s surface area) and significant biodiversity [5], the state of biodiversity in the region has not been evaluated against the Strategic Plan. Without the inclusion of Antarctica and the Southern Ocean, any assessment of the global state of biodiversity in 2020, or by the 2050 end date envisioned by the Strategic Plan, will be incomplete.

Lack of consideration of Antarctica and the Southern Ocean in the context of the Strategic Plan does not imply that the region is free of conservation management. Among several agreements within the Antarctic Treaty System (ATS) [6], the Protocol on Environmental Protection to the Antarctic Treaty (hereafter the Environmental Protocol [7]) and the Convention on the Conservation of Antarctic Marine Living Resources [8] (CAMLR Convention) are explicitly focussed on biodiversity conservation. Moreover, several of the concerns and priorities of the conservation agreements under the ATS are immediately recognizable within the context of the Strategic Plan [5]. However, comprehensive mechanisms do not exist within the Environmental Protocol and the CAMLR Convention for assessing progress against biodiversity conservation targets [9].

An analysis of the state of Antarctic and Southern Ocean biodiversity and its conservation by assessing progress against the Aichi targets would therefore have two major benefits. First, it would offer an opportunity for the assessment and improvement of the state of global biodiversity, as envisaged by the Strategic Plan, to be truly representative of the globe. Second, it would provide the ATS with a way to compare conservation progress in the region with that being made globally and provide a means for future benchmarking. Here, we provide such an analysis to serve these purposes. Other means exist to assess the requirements for and success of biodiversity conservation actions, in a variety of contexts; in some cases, these assessment tools include the identification of specific conservation targets (such as the number and placement of protected areas or specific population trends) [10–12]. We have chosen the current approach because the Strategic Plan forms an encompassing and broadly agreed international strategy for efforts to halt biodiversity loss by 2050, with the clear intent of providing an overarching framework for global biodiversity policy [2].

### Approach

Several approaches for knowledge synthesis to inform biodiversity policy are available [13]. The current assessment was conducted using a combination of empirical evidence and expert knowledge as well as by considering general guidelines for eliciting expert knowledge and conducting biodiversity assessments [14–16] (detailed methods are provided in Supporting Information S1 Text). In brief, 23 experts (the current authors), meeting one or more of four criteria (S1 Text), were convened for a 3-day meeting. Prior to the meeting, all participants were supplied with the objectives of the assessment and key literature that had been compiled in advance, and they were invited to contribute additional relevant evidence to the group, in keeping with similar assessments [17], though with the group being much smaller than for whole-of-field exercises for the region [18]. Contributions were not based on institutional or national representation.

At the meeting, the participants were split into two groups with balanced expertise. Each group completed three tasks, guided by structured worksheets, over the course of the meeting: (1) identify the relevance of each of the 20 Aichi targets to the Antarctic; (2) consider the evidence available to assess the current status for each target for the region; and (3) use this evidence to assess the extent to which the Aichi targets are likely to be realized for the Antarctic region by 2020. For the third task, participants were asked to assign one of five possible trajectories to each target in this task (the same categories used in the 2014 assessment of global biodiversity trends [3]). The full group then met to make a final allocation of one of the five trajectories for each Aichi target. Each decision was assigned a level of confidence (low, medium, or high) based on available evidence and following the Intergovernmental Panel on Climate Change (IPCC) guidelines on uncertainty [19].

The assessment was completed post-meeting by validating the decisions and assigned confidence levels against the primary literature and data sources identified before and during the expert meeting. This evidence was synthesised in support of the decision taken and is reported on here (provided in S1 Text). For much of the Antarctic region, the kinds of indicators that have been applied previously to assess progress towards the Aichi targets [4] are not available. The absence of appropriate quantitative trend indicator data on biodiversity state, pressures, drivers, and response for the region is a recognized challenge that is only now beginning to be addressed [9]. In part, the situation may also reflect the more general difficulty of quantifying progress against the targets [20].

The assessment provided here therefore constitutes a combination of expert knowledge and empirical evidence, weighted towards published evidence for those targets where such evidence was available (S1 Text). Its limitations, compared with other assessments [4], should thus be kept in mind. Moreover, compared with the kinds of reports provided by State Parties to international biodiversity-related agreements, its conclusions are also likely to be more critical. The government ministries responsible for State Party plans and reports have to balance a wide range of domestic and international concerns [21,22]. They may thus be inclined to provide positive reports that focus more on ambition and achievement than on implementation and policy challenges. By contrast, independent expert assessments, while not free from constraints [13,14], are less encumbered.

We document for Antarctica and the Southern Ocean the current status for each of the Aichi targets inclusive of their subsidiary elements, identify the primary supporting data sources, assess trends where data are sufficient to do so, and recognize data gaps that hinder timely management and policy responses. Our assessment complements a recent, comprehensive midterm analysis of progress against these biodiversity targets for the rest of the world [3,4] and provides an evidence base for ATS bodies to contribute to the global assessment of the state and trajectory of biodiversity envisioned for the end of the decade [2]. It also provides a timely input to the Global Assessment on Biodiversity and Ecosystem Services of the Intergovernmental Science-Policy Platform on Biodiversity and Ecosystem Services, which will include an assessment of progress towards achievement of the Aichi targets and relevant Sustainable Development Goals.

### Strategic Goal A: Mainstreaming biodiversity

**Fig 1:**
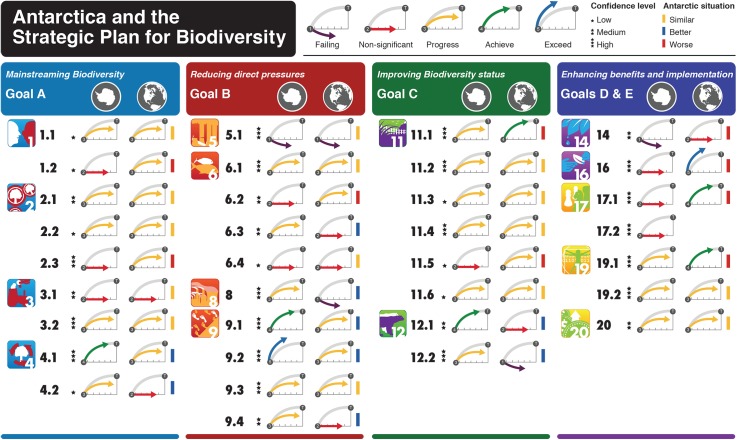
Progress for Antarctica and the Southern Ocean against the Aichi target elements compared with the Global Biodiversity Outlook 4 [3]. Strategic Goal A: (1.1) people are aware of biodiversity values; (1.2) people are aware of steps to conserve biodiversity; (2.1) biodiversity values integrated into development; (2.2) biodiversity values integrated into planning; (2.3) biodiversity values integrated into reporting; (3.1) incentives harmful to biodiversity reduced; (3.2) positive conservation incentives applied; (4.1) plans for sustainable production and consumption; and (4.2) impacts of use within safe ecological limits. Strategic Goal B: (5.1) habitat degradation reduced; (6.1) fish and invertebrate stocks managed sustainably; (6.2) recovery plans and measures in place; (6.3) no significant fishery impacts on threatened species and vulnerable ecosystems; (6.4) fisheries within safe ecological limits; (8) pollutants not detrimental to ecosystem function; (9.1) invasive alien species identified and prioritized; (9.2) pathways identified and prioritized; (9.3) priority species controlled; and (9.4) introduction and establishment of invasive alien species prevented. Strategic Goal C: (11.1) Seventeen percent of terrestrial and inland water areas conserved; (11.2) 10% of coastal and marine areas conserved; (11.3) important biodiversity areas conserved; (11.4) protected areas are ecologically representative; (11.5) protected areas effectively managed; (11.6) protected areas integrated; (12.1) extinction of known threatened species prevented; and (12.2) conservation status of threatened species improved. Strategic Goals D and E: (14) ecosystem services safeguarded; (16) agreement on access and benefit sharing for bioprospecting; (17.1) biodiversity strategy and action plan exists; (17.2) governments include Antarctica and the Southern Ocean in biodiversity strategy and action plans; (19.1) biodiversity knowledge base improved; (19.2) biodiversity knowledge transferred and applied; (20) and financial resources mobilized. Icons created by the Biodiversity Indicators Partnership (BIP) / Secretariat of the Convention on Biological Diversity (SCBD).

For Strategic Goal A, which addresses the underlying causes of biodiversity loss by mainstreaming biodiversity across government and society, Antarctica and the Southern Ocean are largely on a par with global progress (Fig 1). Although variable, awareness of the value of the region’s biodiversity is high and growing [23], but the steps that can be taken to conserve Antarctic biodiversity are not well understood or widely appreciated. Biodiversity values are integrated into development, planning, and reporting (through the ATS), although reporting for the region is not as well developed as it is globally (S1 Text). The reduction of negative incentives, which are harmful to biodiversity, and the development of positive ones are proceeding at a pace comparable with global efforts, although there is scope for further action. For example, subsidies to Southern Ocean fisheries [24] and to whaling continue to be provided.

### Strategic Goal B: Reducing pressures on biodiversity and sustainable use

Progress toward Strategic Goal B is aligned with global advances, but with large variation across the targets (Fig 1). Insufficient effort is being directed to avoid habitat degradation and loss in the region (S1 Text), and the situation is worsening as a consequence of growing science and tourism activity. Likewise, although fisheries management in the region under the CAMLR Convention represents an internationally lauded, ecosystem-based approach, which includes consideration of vulnerable marine ecosystems, it seems unlikely that fishing will be maintained within safe ecological limits by 2020 [25–27]. Although information on which to base such an assessment is sparse, and our confidence in it is therefore low (S1 Text), much uncertainty remains about the ecological impacts of some fisheries [26]. Such uncertainty is further compounded by growing indications of the impacts of climate change on harvested species and their predators and increasing pressure to expand Southern Ocean fisheries [28].

By contrast, considerable headway is being made with the reduction of local pollutant pressures and those from invasive species (Fig 1). For example, much has been done to remediate and reduce local pollution from scientific stations, although pollutants from remote sources, including microplastics [29], continue to influence the region (S1 Text). Southern Ocean seabirds may be at highest risk globally from oceanic plastic pollution [30]. Pressures from invasive alien species (IAS) have been reduced given the high priority accorded to this pressure by the ATS. IAS have been identified and prioritized, pathways are well known, efforts to manage them have been introduced, and eradications have taken place or are being attempted [9,31]. Rapidly changing climates and growing human activity in many parts of the region mean that IAS introduction and establishment, including of pathogens, are, however, unlikely to be prevented entirely by 2020 (S1 Text). Recent assessments have also shown uneven efforts across the Antarctic Treaty Parties in their implementation of IAS-related conservation actions and insufficient attention to surveillance and the potential for intraregional transfers of species [31].

### Strategic Goal C: Safeguarding ecosystems, species, and genetic diversity

Data for this goal are relatively poorly developed for the region (Fig 1). The most comprehensive information is available for protected areas. Because Article 2 of the Environmental Protocol designates Antarctica as a “natural reserve,” the entire continent is often described as highly protected. By contrast, the International Union for Conservation of Nature (IUCN) does not consider the continent a protected area (PA), no overall strategy exists for managing the continent as a PA, and the Environmental Protocol itself provides a mechanism for designating Antarctic Specially Protected Areas (ASPAs). In consequence, the continent as a whole cannot be considered highly protected [32].

**Fig 2:**
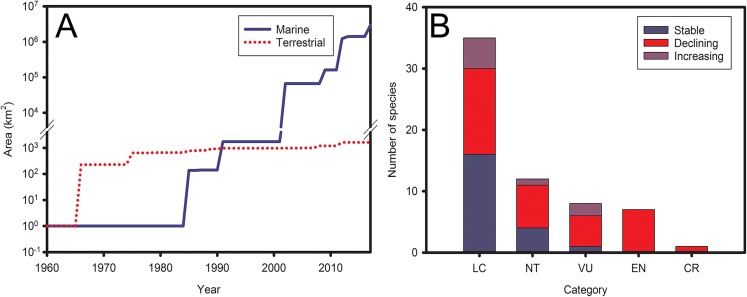
Safeguarding ecosystems and species in Antarctica and the Southern Ocean. (A) Increases in the spatial extent of marine and terrestrial protected areas since the entry into force of the Antarctic Treaty in 1961. Less than 4% of the 45,886 km^2^ ice-free area in continental Antarctica is protected (recognizing that downwards revision of the ice-free area estimate [33] will likely double this percentage). By 2018, 8% of the 35,716 100 km^2^ Southern Ocean CAMLR Convention Area [8] will be under protection. See S1 Table for data. (B) Status trends for the birds of the Antarctic region in each of the 2015 International Union for Conservation of Nature (IUCN) Red List of Threatened Species Categories. LC, least concern; NT, near threatened; VU, vulnerable; EN, endangered; CR, critically endangered. See S2 Table for data.

Both the Antarctic Treaty Consultative Parties and the CAMLR Convention Member States have designated PAs in marine and terrestrial systems. In terrestrial systems, less than 4% of ice-free area falls within ASPAs, though this percentage will likely double given recent downward revisions of the continent’s total ice-free area [33]. Several of the continent’s terrestrial ecoregions remain unprotected [34], and the rate of designation has slowed (Fig 2A). PA designation for marine systems has followed a more positive trajectory. Until recently, only a single large marine protected area (MPA) had been declared south of 60°S (the South Orkney Islands MPA of 94,000 km^2^), with nine ASPAs of smaller size (all less than 1,000 km^2^) being entirely or mostly marine. Three other large MPAs had been declared north of 60°S in the CAMLR Convention Area. At its 2016 meeting, however, the Commission for the Conservation of Antarctic Marine Living Resources (CCAMLR) designated a further 1.55 million km^2^ in the Ross Sea as an MPA, to come into force in December 2017 (S1 Text). Together, PAs in the Southern Ocean will thus, by late 2017, comprise 2.97 million km^2^, approximately 8% of the 35.7 million km^2^ covered by the CAMLR Convention Area. Additional MPA proposals submitted to the CCAMLR in recent years would, if adopted, bring the total area under protection close to 10%. Progress has thus been better from a marine perspective than from a terrestrial one (Fig 2A), acknowledging the protracted nature of MPA negotiations at the CCAMLR [28]. Area targets are thus progressing well but unlikely to be reached by 2020; it is also recognized that for both terrestrial and marine systems, area protection alone may be insufficient [35] and that the PA system is not yet ecologically representative or representative of all areas important for biodiversity (S1 Text), nor are many of the areas effectively managed for biodiversity conservation [34].

Antarctica is the only continent from which anthropogenic extinctions have yet to be recorded [36]. Given current conservation measures, the extinction of known threatened species is also unlikely by 2020. Thereafter, the situation is less clear (S1 Text). Much information is available on the status of seabirds, seals, and cetaceans via the IUCN Red List of Threatened Species and through the work of the Agreement on the Conservation of Albatrosses and Petrels. In many cases, however, comprehensive assessments and analyses of trends are challenging given the remoteness of the region, which precludes effective data gathering, a situation being addressed in part through innovative satellite remote sensing [5]. The available information indicates a decline in the status of many species (Fig 2B), with climate change and IAS at sub-Antarctic breeding localities being major drivers of change (S1 Text). Conservation actions to remedy the situation include extensive eradication programs at or planned for many of these islands. By contrast, few formal assessments of status or trends exist for other species (especially plants and invertebrates), despite the fact that many are endemic to single locations.

### Strategic Goal D: Benefits from biodiversity and ecosystem services

The benefits from Antarctic and Southern Ocean biodiversity and ecosystem services are realized mainly via the vast and productive Southern Ocean ecosystem given its significance in the Earth system and especially for CO_2_ sequestration [37]. Increases in atmospheric CO_2_, ocean acidification, and substantial changes to ocean and atmospheric circulation patterns and sea ice will move the Southern Ocean away from a target concerned with restoring and safeguarding ecosystem services (Fig 1). New modelling has also indicated substantial changes may be expected to Antarctic ice sheets before the turn of the century, with considerable global consequences [38]. This situation is different from assessments for the rest of the globe, where the outlook is for no net worsening of the current situation.

The ATS currently has no agreed mechanisms for the management of bioprospecting, the route through which genetic resources are exploited in the region (S1 Text). Existing conservation arrangements preclude population-level impacts, but benefit sharing remains problematic and is unlikely to be addressed by 2020. The Antarctic Treaty Consultative Parties have adopted only two hortatory resolutions concerning bioprospecting, despite many years of discussion of the matter. Given the unique governance arrangements in the region under the ATS, the CBD’s Nagoya Protocol on Access to Genetic Resources and the Fair and Equitable Sharing of Benefits Arising from Their Utilization does not strictly apply [39].

### Strategic Goal E: Participatory planning

Implementation of Strategic Goal E is not well developed for the region (Fig 1). Agreements such as the Environmental Protocol and the CAMLR Convention do provide a forum for such planning, and joint work among ATS bodies has taken place on several occasions (S1 Text). Yet, no integrated and comprehensive biodiversity strategy or plan exists for the region. By contrast, knowledge management and capacity building are consistent with global trends, as a consequence of growing information on biodiversity and its conservation [5], and a suite of new activities to communicate this information to decision makers [40]. Notable, however, is the absence of specific strategies for the collection of information that would enable better assessment and management of trends in the status of biodiversity (see discussion in [41]).

## Conclusions

Overall, our assessment suggests that the biodiversity prospects for Antarctica and the Southern Ocean for 2020, and beyond to 2050, are similar to those for the rest of the planet (Fig 1). Such a conclusion will strike many as controversial, especially if selected, individual comparisons are made, such as between global habitat destruction and the impression of what is happening on the Antarctic continent. Indeed, in many ways the Antarctic region is often considered a gold standard for conservation management (see discussion in [42–44]). Our evidence-based assessment suggests, however, that the current situation is not quite living up to such a view.

By contrast, the ATS agreements offer an unparalleled opportunity to improve matters over the next 5 years. They lend themselves to effective action [6], and there is wide support from governments [45], industry [46], and the public for conservation of the region. The development of an integrated biodiversity strategy and action plan for Antarctica and the Southern Ocean would deliver a roadmap to harness this support. Along with deployment of new tools to monitor and manage biodiversity [5,41,47], its implementation could improve the outlook for the region, to the benefit of all.

## Supporting information

S1 Fig Mentions of biodiversity (and stem words ‘biodiv*) in the reports of the CCAMLR and CCAMLR Scientific Committee from 1982 until 2015. Data were extracted by counting mentions of the words in these reports and summing for the combined number. These reports are available online at www.ccamlr.org.
(TIF)

S1 Table The spatial extent and years of designation of marine and terrestrial protected areas. Data for the table were obtained from the Antarctic Treaty (www.ats.aq) polygons for these areas, the management plans for the marine protected areas, and from CCAMLR (www.ccamlr.org). The data are current as of November 2016.
(XLSX)

S2 Table Data on the Red List Category and Trends for Antarctic and Southern Ocean birds. Extracted from the IUCN Red List of Threatened Species in July 2016.
(XLSX)

S3 Table International Agreements to which Antarctic Treaty Parties are party and the status of their National Biodiversity Strategy and Action Plans under the Convention on Biological Diversity. Data current as of September 2016.
(XLSX)

S1 Text Methods and evidence supporting the assessment outcome. This section provides a full description of the methods used and comprehensive information supporting the assessment presented in the manuscript.
(DOCX)

## Abbreviations

ASPA: Antarctic Specially Protected Area
ATS: Antarctic Treaty System
BIP: Biodiversity Indicators Partnership
CAMLR Convention: Convention on the Conservation of Antarctic Marine Living Resources
CBD: Convention on Biological Diversity
CCAMLR: Commission for the Conservation of Antarctic Marine Living Resources
IAS: invasive alien species
IPCC: Intergovernmental Panel on Climate Change
IUCN: International Union for Conservation of Nature
MPA: marine protected area
PA: protected area
SCBD: Secretariat of the Convention on Biological Diversity
