# Supplementary figures and images for: Antarctica and the strategic plan for biodiversity
Source: PLoS Biol. 2017 Mar 28;15(3):e2001656. doi: 10.1371/journal.pbio.2001656 (PMC5369689; doi:10.1371/journal.pbio.2001656)

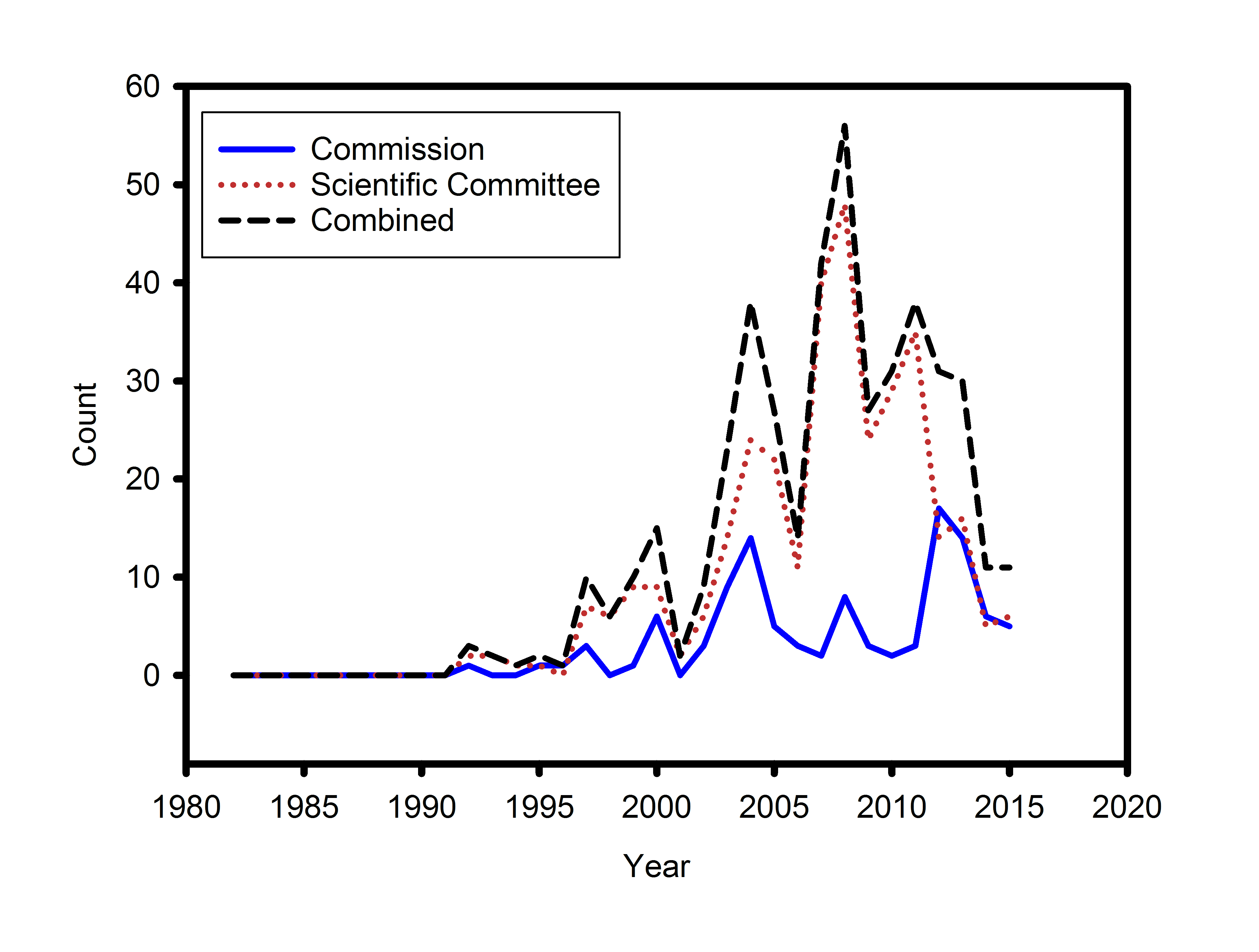

Supplement: S1 Fig — Data were extracted by counting mentions of the words in these reports and summing for the combined number. These reports are available online at www.ccamlr.org. (TIF) [file pbio.2001656.s001.TIF]
