# Supplementary material for: Antarctica and the strategic plan for biodiversity
Source: PLoS Biol. 2017 Mar 28;15(3):e2001656. doi: 10.1371/journal.pbio.2001656 (PMC5369689; doi:10.1371/journal.pbio.2001656)
Supplement: S1 Text — This section provides a full description of the methods used and comprehensive information supporting the assessment presented in the manuscript. (DOCX) [file pbio.2001656.s005.docx]

**Chown S1 Text**

Antarctica and the Strategic Plan for Biodiversity

**Steven L. Chown^1*†^, Cassandra M. Brooks^2^, Aleks Terauds^3^, Céline Le Bohec^4,5,6^, Celine van Klaveren-Impagliazzo^7^, Jason D. Whittington^4,8^, Stuart H.M. Butchart^9,10^, Bernard W.T. Coetzee^1^, Ben Collen^11^, Peter Convey^12^, Kevin J. Gaston^13^, Neil Gilbert^14^, Mike Gill^15^, Robert Höft^16^, Sam Johnston^17^, Mahlon C. Kennicutt II^18^, Hannah J. Kriesell^4^, Yvon Le Maho^4,5,6^, Heather J. Lynch^19^, Maria Palomares^20^, Roser Puig-Marcó^21^, Peter Stoett^22^, Melodie A. McGeoch^1^**

**1** School of Biological Sciences, Monash University, Melbourne, Australia, **2** School of Earth, Energy and Environmental Sciences, Stanford University, Stanford, United States of America, **3** Australian Antarctic Division, Department of the Environment and Energy, Kingston, Australia, **4** Centre Scientifique de Monaco (CSM), Département de Biologie Polaire, Monaco, Principality of Monaco, **5** Université de Strasbourg (UdS), Centre National de la Recherche Scientifique (CNRS), Institut Pluridisciplinaire Hubert Curien (IPHC) UMR 7178, Strasbourg, France, **6** Laboratoire International Associé LIA 647 *BioSensib* (CSM-CNRS-UdS), Monaco, Principality of Monaco, **7** Direction des Affaires Internationales, Département des Relations Extérieures et de la Coopération, Ministère d’État, Gouvernement Princier, Principauté de Monaco, **8** Centre for Ecological and Evolutionary Synthesis, Department of Biosciences, University of Oslo, Norway, **9** BirdLife International, Cambridge, United Kingdom, **10** Department of Zoology, University of Cambridge, Cambridge, United Kingdom, **11** Centre for Biodiversity & Environment Research, Department of Genetics, Evolution & Environment, University College London, London, United Kingdom, **12** British Antarctic Survey, NERC, Cambridge, United Kingdom, **13** Environment & Sustainability Institute, University of Exeter, Penryn, United Kingdom, **14** Antarctica New Zealand, Christchurch, New Zealand, **15** Polar Knowledge Canada, Government of Canada, Ottawa, Canada, **16** Secretariat of the Convention on Biological Diversity, Montreal, Canada, **17** Melbourne Law School, The University of Melbourne, Melbourne, Australia, **18** Texas A&M University, College Station, United States of America, **19** Department of Ecology & Evolution, Stony Brook University, Stony Brook, United States of America, **20** Sea Around Us, Global Fisheries Cluster, Institute for the Oceans and Fisheries, University of British Columbia, Vancouver, Canada, **21** Faculty of Law, University of Barcelona, Barcelona, Spain, **22** Loyola Sustainability Research Centre, Concordia University, Montreal, Canada

**Methods and evidence supporting the assessment outcome.**

**Materials and Methods**

*Geographic coverage*

The assessments provided here apply to the geographic area covered by the Antarctic Treaty (land and ocean south of 60°S) and by the Commission on the Conservation of Antarctic Marine Living Resources (CCAMLR) Convention Area. The CAMLR Convention Area is defined as the area south of the Antarctic Convergence (or Antarctic Polar Front), though in practice it extends in some places to the north of the Front, and in others does not quite reach it [1]. As a consequence of its definition, the CAMLR Convention Area includes several sub-Antarctic islands, which are each sovereign territory of a particular nation (Australia, France, Norway, South Africa, and, though disputed, the United Kingdom), including the exclusive economic zones (EEZs) around them (for a review of these islands see Ref. 2). Given that some species breed on the islands, but forage in the seas around them (such as albatrosses, petrels, penguins and seals), our assessments by necessity sometimes blur the geopolitical distinction between the sovereign areas of the sub-Antarctic islands, and the Antarctic Treaty and CAMLR Convention areas. Nonetheless, we have not set out here specifically to assess the situation for the sub-Antarctic islands and their EEZs because they are necessarily incorporated, at least legally, into country-specific assessments made by Parties to the Convention on Biological Diversity (CBD). In consequence, we consider these islands only where it is necessary and then mostly from a marine perspective or where considering the breeding areas of seabirds and seals. Where we make further reference to the terrestrial areas of these islands we do so only for comparative purposes. In adopting this approach, we recognize that the geopolitical and biological division of the Antarctic region has been the subject of much discussion [3,4], and that Article II of the Protocol on Environmental Protection to the Antarctic Treaty (hereafter the Environmental Protocol) recognizes the ‘dependent and associated ecosystems’ of Antarctica without defining what these are [5].

*Assessment approach*

The assessment was conducted using a combination of empirical evidence and expert knowledge, and considering general guidelines for eliciting expert knowledge and conducting biodiversity assessments [6-8]. The study context and justification are outlined in the main body of the work, and parallel the justification for the Strategic Plan for Biodiversity 2011-2020 (hereafter the Strategic Plan) [9], with the context limited to the Antarctic as described in the previous section.

***Expert engagement*.** The most recent and synthetic primary literature directly relevant to the Antarctic context and the 20 Aichi Biodiversity Targets (hereafter Aichi targets) were compiled. A group of experts was then identified that met one or more of the following criteria: (i) were lead authors of this relevant primary literature, (ii) had specific and complementary expertise in areas relevant to the Antarctic and one or more of the Aichi targets, (iii) had substantial experience in conducting biodiversity assessments, including those conducted for the purpose of reporting on biodiversity targets, (iv) were environmental and biodiversity legislation, policy or management experts, including with intimate familiarity with the Antarctic Treaty System (ATS) or the CBD. Twenty-three experts were finally included and represented expertise across the full suite of Aichi targets relevant to the region, with two or more experts for most Targets (most participants had expertise that spanned 2-4 targets).

***Elicitation process*.** Prior to holding a three-day meeting of the expert group, all participants were supplied with the objectives of the assessment, key literature and were invited to contribute additional relevant evidence to the group in advance of the meeting. Participants were requested to familiarise themselves in advance of the meeting with the assessment objectives, Strategic Plan and Aichi targets, background information on environmental governance of the Antarctic and the relevant literature provided. The meeting started with general orientation (e.g., geographic scope of region to be considered), discussion of the assessment context and justification, discussion of best practice principles of expert judgement and the potential biases involved in expert elicitation processes [7].

The group was split into two with the diversity of expertise balanced as far as possible across the groups. Each group completed three tasks, guided by structured worksheets, over the course of the meeting:

(1) Identifying the relevance of each of the 20 Aichi targets to the Antarctic (yes or no), suggestions for alternative wording where appropriate so that the target became clearly relevant to the region (in some cases it was possible to make the targets more precise or specific given the narrower geographic context), allocating an importance ranking to each target (low, moderate, high; importance to achieving the overarching strategic goals for biodiversity).

(2) Considering the evidence available to assess the current status for each target for the region (with reference to data sources and key publications), including the identification and description of evidence available and a rating of the adequacy of evidence for each target as comprehensive, good, moderate, poor or none.

(3) An assessment of the extent to which the Aichi targets (as modified for the Antarctic) are likely to be realized for the Antarctic region by 2020 (the date set by the Strategic Plan for target achievement [9], and in some cases consideration of a 2050 endpoint, the date set for an end to biodiversity loss [9]). Participants were asked to assign one of five possible trajectories to each target in this task (the same five trajectory status categories used to communicate the current status of progress towards targets used in the 2014 assessment of global biodiversity trends, policy scenarios and key actions [9]). The trajectory status categories were (1) on track to exceed target, (2) on track to achieve target, (3) progress towards target but at an insufficient rate, (4) no significant overall progress, (5) moving away from target. These were represented using the same trajectory icons used in the target dashboard in GBO4 [9].

The final step of reaching a provisional agreement on the assignment of current trajectory status was conducted with the full group, explicitly reporting on the decisions of the two sub-groups, followed by discussion and final allocation of one of the five trajectories. Each decision was assigned a level of confidence (low, medium or high) based on available evidence and following the Intergovernmental Panel on Climate Change (IPCC) guidelines regarding uncertainty [10].

***Knowledge assessment.*** The assessment was completed post-meeting by validating the decisions and assigned confidence levels against the primary literature and data sources identified before and during the expert meeting. This evidence was synthesised in support of the decision taken and is reported on here. For much of the Antarctic region, the kinds of indicators that have been applied previously to assessment of the progress towards the Aichi targets [11] are not available. The absence of appropriate quantitative trend indicator data on biodiversity state, pressures, drivers and response for the region is a recognized challenge that is only now beginning to be addressed [12]. In part, the situation may also reflect the more general difficulty of quantifying progress against the targets [13,14]. Thus, we used evidence, including qualitative assessments, from a range of primary sources and systematic reviews to make our assessments. In each case, we indicate these sources, note where information is uncertain or unavailable, and reach a conclusion based on this information. The final outcome therefore constitutes a hybrid outcome involving qualitative and quantitative knowledge, heavily weighted towards published evidence for those targets where such evidence was available.

**Evidence Supporting the Assessment Outcomes**

**Strategic Goal A. Address the underlying causes of biodiversity loss by mainstreaming biodiversity across government and society**

**Target 1 People are aware of biodiversity and steps they can take to conserve it**

*1.1 People are aware of the values of biodiversity*

Awareness of Antarctic biodiversity is variable according to formal surveys. For example, a survey of young Argentinians indicated that knowledge of environmental problems in the region was low [15]. In Malaysia, for secondary school students, knowledge of biodiversity-related problems in the region was better developed [16]. Overall it appears that appreciation of the environmental or wilderness, rather than strictly biodiversity, value of the region is high and perhaps growing [17,18], though surveys are relatively limited in number and scope.

The full extent of marine biodiversity is becoming more widely appreciated as a consequence of a wide range of popular written and visual media on the region, and especially the Ross Sea [19]. Substantial media and social media campaigns by a range of government agencies and non-governmental organizations (NGOs) (e.g., the Antarctic and Southern Ocean Coalition, ASOC) promote Antarctic marine diversity. The tourism industry, through its industry association, the International Association of Antarctica Tour Operators (IAATO), also has substantial education campaigns on tourist cruises to the continent, usually in the form of briefings and expert talks on wildlife likely to be encountered [20]. The extent and richness of terrestrial diversity are more poorly known, largely because this diversity does not include charismatic species. Nonetheless, media interest in the unusual ecosystems and species of the terrestrial Antarctic is growing [21]. Likewise, the significance of terrestrial biodiversity is promoted through IAATO, specifically in a diversifying variety of multimedia products about the value of, and threats to, terrestrial Antarctic diversity [22]. Owing to the range of agencies and organizations involved in promoting Antarctic biodiversity, awareness of its values is growing, but is unlikely to be universal within the Target’s 2020 timeframe.

*1.2 People are aware of the steps they can take to conserve and sustainably use biodiversity*

Given the limited number of formal surveys of environmental values in the region (reviewed most recently in Ref. 18), and their indication that knowledge of biodiversity-related values is variable, little evidence is available about progress towards this Target. Given the extant evidence it seems likely that awareness of steps to conserve Antarctic biodiversity will not be attained by 2020.

**Target 2 Biodiversity values incorporated into planning, accounting and reporting**

*2.1 Biodiversity values integrated into national and local development strategies*

A coherent, comprehensive strategy for Antarctic biodiversity does not yet exist. Elements thereof are captured in the Protocol on Environmental Protection to the Antarctic Treaty [23], the Convention on the Conservation of Antarctic Marine Living Resources [24], and various measures, decisions and resolutions of the Antarctic Treaty Consultative Meetings (ATCMs) and meetings of the CCAMLR (see discussion in Ref. 25 and further summary in Ref. 26). Other agreements include biodiversity planning for the region, such as the Agreement on the Conservation of Albatrosses and Petrels (ACAP), and the Convention on the Conservation of Antarctic Seals (CCAS) [26]. Although the CCAS depository government (United Kingdom) continues to report seal catches, CCAS is largely not operational because commercial sealing is not underway in the region. The International Union for Conservation of Nature (IUCN) and the Scientific Committee on Antarctic Research (SCAR) published a strategy for Antarctic conservation in the early 1990s [27]. Given that it predated the Environmental Protocol, many of its major recommendations could have been taken up within Environmental Protocol, but the IUCN and the SCAR neglected to send the document to the appropriate ATCM. The strategy apparently failed to have any substantive influence on the Antarctic Treaty Consultative Parties (ATCPs) [28].

Few formal development strategies for local terrestrial areas of the Antarctic are available. Some stations do take biodiversity and impacts on biodiversity into account in development strategies [29], though few formal plans are readily available in the public domain. Antarctic Specially Managed Areas (ASMAs), which may be designated under the auspices of the Environmental Protocol (Annex V on Area Protection and Management), constitute a further avenue for formal development strategies within the region. A clear example, including biodiversity values, is provided by the Management Plan for ASMA No.2 McMurdo Dry Valleys, Southern Victoria Land [30].

While the CAMLR Convention does not explicitly mention biodiversity, it does mandate conservation of the Southern Ocean marine ecosystem, which includes components of biodiversity [1]. In managing the commercial harvest of fish species and krill under a precautionary and ecosystem-based mandate, the CCAMLR effectively considers biodiversity in its management rules. Accordingly, fisheries cannot be developed without a thorough and analytical consideration of impacts on the species harvested and the greater ecosystem. However, due to lack of data, which in part results from the difficulty of conducting research during much of the year (e.g., heavy ice cover in winter months) (see also discussion in Ref. 31) and lack of financial resources/capacity, the ecosystem impacts of most CCAMLR fisheries remain unknown and poorly quantified. In an effort to conserve biodiversity more effectively and explicitly, especially given the threat of climate change, the CCAMLR has been working towards the establishment of Marine Protected Areas (MPAs), with a major recent breakthrough [32], despite stalled efforts in previous years [33,34] (see also see Target 11.2 below).

Overall, while progress is being made, including the development of an Antarctic Conservation Strategy for the 21^st^ Century by SCAR and partners [35], it appears that this Target Element will not be fully attained by 2020 or by 2050 without significant additional effort and resourcing.

*2.2 Biodiversity values integrated into national and local planning processes*

Local development strategies, incorporated into Environmental Impact Assessments and into Antarctic Specially Protected Area (ASPA) Management Plans, both mandated by the Environmental Protocol (Annexes I and V, respectively), include biodiversity values. Similarly, biodiversity values are incorporated into CCAMLR Conservation Measures, such as protection for Vulnerable Marine Ecosystems (VMEs) [36], prohibitions on gillnets and bottom trawling, rules to mitigate incidental seabird catch and fish by-catch, and general environmental measures [37,38].

In a terrestrial setting, some doubt has been raised about the efficacy of ASPA Management Plans because they are frequently not ecologically realistic [39]. For example, strict measures to reduce the transfer of propagules of invasive alien species into a given ASPA make no sense if those same restrictions are not applied to the remaining ice-free area of which the ASPA is part. Likewise, not a single major development activity (such as the construction of a new station, or a hard rock runway) appears to have been substantially modified, let alone halted, as a result of conducting a comprehensive environmental evaluation, despite these potential outcomes being mandatory considerations within Annex I to the Environmental Protocol [40].

Given that the economic activities in the Antarctic region are largely science, fishing and tourism, all of which are considered from the perspective of their environmental, including biodiversity, impacts by the Committee for Environmental Protection (CEP) and the CCAMLR, it may be argued that region-wide planning which incorporates biodiversity values is undertaken. Likewise, the provisions of the various ATS agreements, and the various recommendations made at regular meetings of the Parties to those Agreements (usually in the form of Measures and Resolutions, with Decisions being about internal organizational matters), are given effect to through national legislation of the Parties [26]. This domestic legislation is frequently listed in National Biodiversity Strategy and Action Plans (including for several of the Antarctic Treaty Consultative Parties such as Argentina, Australia, Belgium, France, Japan, New Zealand, South Africa, and the non-consultative party Romania). Nonetheless, very little further detail on Antarctica and the Southern Ocean (except for areas of national jurisdiction) is provided.

Thus, whilst biodiversity values are incorporated into national and local planning, and activity to address this Target Element appears to be increasing, it is unlikely to be fully met by 2020.

*2.3 Biodiversity values incorporated into reporting systems*

Biodiversity values are incorporated into reporting to both the CCAMLR and the CEP given the mandates set by the CAMLR Convention and the Environmental Protocol. Since the commencement of the CEP meetings, a wide range of Working and Information Papers concerning biodiversity values has been presented annually either by Party members of the CEP or by NGOs (see Ref. 41 and for a full listing the Antarctic Treaty Secretariat meetings page [42]). Similarly, the discussion of biodiversity-related matters has increased over the years at CCAMLR (S1 Fig). Nonetheless, as pointed out in the CCAMLR Performance Review [43], ‘…the status of many by-catch species is unknown or poorly known, the broader ecosystem monitoring of biodiversity and dependent predators is not well connected to management decision-making…’. Some steps are being taken to enhance current understanding and provide a baseline for such reporting [44,45], but reporting of changes to biodiversity values is generally not undertaken in a systematic manner at either CCAMLR meetings or at the CEP, even on a periodic basis. In consequence, no overall progress toward or retreat from achieving this Target Element by 2020 has been made.

**Target 3 Incentives, including subsidies, harmful to biodiversity are eliminated, phased out or reformed, and positive incentives developed**

*3.1 Incentives, including subsidies, harmful to biodiversity, eliminated, phased out or reformed in order to minimize or avoid harmful impacts*

Two forms of incentives were considered here. The first is the geopolitical pressure that exists for nations to continue to have a presence in Antarctica. The Antarctic Treaty provides that Consultative Party (or “voting”) status at ATCMs is achieved by demonstrating interest in Antarctica through the conduct of substantial scientific research activity there. The Treaty provides that such demonstration can be achieved through the establishment of a scientific station or the despatch of a scientific expedition [25,26,46]. Typically, ATCPs have interpreted this to mean the construction and occupation of government-funded stations on the continent, with the number of stations continuing to increase [47]. Only the Netherlands has so far managed to retain its scientific presence without a full station presence, although recently constructing and maintaining a laboratory with the United Kingdom at Rothera Station [48]. Over the past decade, several new stations have been proposed [49] or established [50-53] and arguments have been made [54] that such stations should be shared to reduce environmental impacts, especially on the limited ice-free areas that are usually preferred for stations [55,56], and to reduce logistic costs. To date station sharing is uncommon (but examples do exist, e.g., Concordia by France and Italy; Rothera by the U.K. and Netherlands; Carlini by Argentina and Germany). Instead, new stations and other facilities continue to be constructed, indicating that little is being done to reform these practises. It should be noted, however, that considerable international collaboration is the norm in much Antarctic science, and scientists from several nations are typically hosted at many stations in any one season. One argument that has been made in mitigation of the criticism of continued station building is that scientific demand is now so high [31,57,58] that stations are at capacity and new stations are required. That demand could, however, be met by investment in extending current station capacity jointly, yet this is rarely done. Rather the geopolitical and perhaps economic imperatives for national stations remain significant [59,60].

The second form of incentive is incentives or subsidies to fisheries in the region. Information here is not readily available for the national fisheries operating in the Southern Ocean. Prior to the breakup of the Soviet Union, government subsidies enabled its fishing fleet to expand into the Southern Ocean [61] with considerable impacts on fish stocks in the CAMLR Convention Area [62]. Those subsidies largely disappeared with the collapse of the Soviet Union and Southern Ocean fisheries are now dominated by other nations [33,63]. Which of the currently operating national fisheries receive subsidies remains unclear. Subsidies to krill fishing have apparently ceased in South Korea [59]. China has an explicit program through its Ministry of Agriculture to support Southern Ocean fishing, known as the Antarctic resource exploration program, which received a portion of its US$ 6.5 billion subsidy to fisheries in 2013 [64]. China’s Twelfth Five-year Fisheries Plan will see expansion of the scope of Antarctic marine living resources surveys and expeditions [64].

In the case of whaling, the only programme operating in the Antarctic, given the 2015 catch of 300 Minke Whales by the Japanese fleet [65], does so at a significant loss financially, with subsidies and low interest loans from the Japanese government [4,66].

Overall, although some subsidies have been discontinued, others seem to be continuing, with no net change in this Target Element expected by 2020, though recognizing that detailed information on incentives and subsidies is not widely available.

*3.2 Positive incentives for conservation and sustainable use of biodiversity developed and applied*

As is the case with the previous Target Element, little information is available on positive incentives for conservation and sustainable use. Moreover, assessing positive incentives might be considered geopolitically complicated in the Antarctic. The signing of the Antarctic Treaty suspended territorial claims to the continent and surrounding waters, although the Antarctic, and the resources it harbours, remain contested to some degree [67]. While conservation is an important goal of many of the ATCPs and CCAMLR Member States, those most active in this arena, especially where it comes to the establishment of ASPAs and proposing MPAs, are in the majority those States with historic claims [68,69]. This has led to accusations that area protection on land and sea is being used as a means to reinforce national strategic interests, rather than conservation [68].

In the fisheries realm, positive incentives exist for sustainable use through Marine Stewardship Council (MSC) certifications and CCAMLR Conservation Measures. Several CCAMLR krill and toothfish fisheries are currently MSC certified [70,71], incentivising sustainable harvest through greater market access. However, the effectiveness of MSC certification in leading to more sustainable fishing practices has been heavily scrutinized, particularly for Southern Ocean fisheries [72]. The CCAMLR has been successful at de-incentivizing illegal, unregulated and unreported (IUU) fishing through implementing strict measures for traceability (through a Catch Documentation Scheme), sanctioning (through IUU vessel black-list), surveillance (through Vessel Monitoring Systems) and other rules [73,74]. While some progress towards this Target Element is being made, incentives for conservation and sustainable use are limited and complex. Thus this target is unlikely to be met by 2020.

**Target 4 Use of natural resources kept within safe ecological limits**

*4.1 Governments, business and stakeholders at all levels have taken steps to achieve, or have implemented, plans for sustainable production and consumption*

The existence of the Environmental Protocol (since its entry into force in 1998) and the CAMLR Convention (since 1982; 24) and the operation of these agreements indicate that government, business (tourism and fishing) and stakeholders (conservation NGOs such as ASOC) have taken steps to achieve sustainable consumption in the region. While these agreements face significant challenges [47,62,75], their ongoing existence, and the affirmation that conservation in the region is of considerable importance to the ATCPs [76] suggest that this target will be met. Moreover, recent industry developments also indicate that business is organising to contribute to sustainable consumption. Since 1991, IAATO [77] has self-organized the tourism industry to do so (see also discussion in Ref. 78), and others are following suit. Examples include the Coalition of Legal Toothfish Operators (COLTO, <http://www.colto.org>) and the Association of Responsible Krill Harvesting Companies (ARK, <http://www.ark-krill.org/>). In consequence, progress towards this Target Element is being made, and it seems likely to be reached by 2020, depending to some extent on activities of those not represented by IAATO and on further developments in the fishing sector.

*4.2 Impacts of use of natural resources kept well within safe ecological limits*

Quantifying ‘safe ecological limits’ is difficult under any circumstances [13,14], but may be especially challenging in the Antarctic due to the impacts of climate change and the general lack of data and ecological indicators [12,47,79]. The Antarctic is one of the most rapidly changing environments on Earth, with dramatic fluctuations in temperature, currents and sea ice [80,81]. In the western Antarctic Peninsula, these environmental changes have driven declines in primary productivity [82], shifts in algal community composition [83], and, apparently, increases in salps, and some changes in populations of Antarctic krill [84,85]. Despite these recorded changes in the well-studied Antarctic Peninsula, much uncertainty remains about the ecological consequences of climate change across the Antarctic and Southern Ocean [86].

The increasing environmental pressure of Antarctic research station building and fishing [47,87,88] will likely compound the impacts of climate change. With declines in krill and reductions in ice, fishing vessels are increasingly encroaching into penguin foraging grounds [89,90]. Whether or not toothfish fisheries are operating within ‘safe ecological limits’ remains intensely debated, with some scientists insisting that insufficient data exist to determine the ecological impacts of toothfish fisheries, particularly in the Ross Sea [91]. In general, Antarctic fisheries have proven not to be highly productive, and slow to recover, making them vulnerable to overfishing [92]. Moreover, a general paucity of environmental and ecological data is the case for much of the Antarctic due to lack of technological, scientific and financial capacity [93]. While steps are being taken to meet this target (as referred to under 4.1 above), and through the Environmental Protocol and CCAMLR, this Target Element is unlikely to be met. The low confidence indicates the uncertainty associated with this adjudication.

**Strategic Goal B. Reduce the direct pressures on biodiversity and promote sustainable use**

**Target 5 Loss of habitats halved and degradation and fragmentation reduced**

*5.1 The loss of all habitats is at least halved and where feasible brought close to zero, degradation is significantly reduced*

In the terrestrial context, the ice-free areas of Antarctica are the main focus. These ice-free areas constitute < 0.4% (45 886 km^2^) of the total continental surface area (*ca*. 14 million km^2^) [94,95]. Nonetheless, much activity in the form of science, research stations (*ca*. 80% in coastal areas), and tourism is concentrated in these areas, as is much biodiversity [47,96]. Not only is activity in the form of research station construction and numbers of science personnel increasing [12], but so too is tourism, following a temporary lull associated with the Global Financial Crisis and a change in fuel requirements [97-99]. Several reports have suggested that ASPA Management Plan aims, restrictions or measures are frequently either inadequate or not adhered to [39,100]. Moreover, general habitat degradation is commonplace, through trampling by individuals, vehicle-associated damage, and other activities at stations [100-104]. The significance of general terrestrial habitat degradation is not widely appreciated from a conservation action perspective, judging by submissions to the CEP, with the exception of human trampling which is widely mentioned in ASPA Management Plans and which has been raised in specific Working Papers discussed at the CEP meetings [105] and in a specific Resolution [106] concerning efforts to minimize trampling effects on moss beds (see Target 8 below for consideration of local pollution). Discussion of environmental remediation and repair [107,108], which remains a standing item on the CEP five-year workplan, can, however, be considered equivalent to a general concern about habitat degradation, although at its most recent meeting the CEP devoted no discussion to the matter [105]. The absence of general concern for habitat degradation and plans to reduce it, along with an increase in all forms of activity (including a diversification of tourism [109]) suggests that, from a terrestrial perspective, matters are getting worse, rather than better.

In marine systems, iceberg scour is common in near-shore habitats [110], thus habitat disturbance of this form is an integral part of the ecosystem. In the CAMLR Convention Area, benthic trawling is effectively prohibited [38], with the exception of waters that fall within national jurisdiction. Disturbance to the seabed is mostly from scientific research trawling [111] and from limited commercial demersal trawling, now largely restricted to icefish operations around Heard Island and the McDonald Islands [112,113]. Benthic longlining is undertaken in some areas where vessels target toothfish, but the CCAMLR has conservation measures in place to reduce the impacts of longline fishing activities especially in Vulnerable Marine Ecosystems [38].

Overall, particularly in the terrestrial environment, evidence suggests that habitat loss will continue and that this target will not be met in the Antarctic by 2020.

*5.2 Fragmentation is significantly reduced*

Reduction in fragmentation was not evaluated. Terrestrial Antarctic habitats are highly fragmented in the absence of any human intervention. How this fragmentation has influenced patterns of genetic diversity is poorly understood, though it is clear that substantial spatial structuring is present [114,115]. Such structuring is apparent even where habitats appear physically contiguous, such as in the Dry Valleys of the Ross Sea region [116]. Likewise, spatial genetic structure has also been found in a variety of marine groups [114,117]. A major concern raised in the terrestrial context is the artificial connectivity that might be promoted by the transfer of indigenous species between fragmented locations on the continent, so reducing genetic heterogeneity [12,39].

**Target 6 Fish and invertebrate stocks managed and harvested sustainably**

*6.1 All fish and invertebrate stocks are managed and harvested sustainably, legally and applying ecosystem based approaches*

The Convention on the Conservation of Antarctic Marine Living Resources entered into force in 1982 [24] with the primary objective of conserving Antarctica’s marine living resources, but conservation was defined to include ‘rational use.’ Rational use permits fishing in the CAMLR Convention Area, but mandates a strict, precautionary, ecosystem approach to management that must consider the impacts of any fishery on the related and dependent species as well as the environment [24]. The CAMLR Convention is widely regarded as an early, progressive, ecosystem-based approach to managing living resources and CCAMLR is seen as a leader among other high seas regional fisheries management organizations [1,62]. The CCAMLR, comprising 24 Member States and the European Union, is responsible for managing all harvested fish and invertebrate stocks in the Southern Ocean.

Many studies have considered the complexities of the multi-species (ecosystem-based) approach adopted by CCAMLR, the efficacy of the Convention and Commission, and the prognosis for Southern Ocean fisheries [1,62,75,118,119]. The CCAMLR has been remarkably effective in reducing IUU fishing, which was widespread in the late 20^th^ Century [74,120], reducing seabird mortality associated with several aspects of the Southern Ocean fishery [121], and has also been celebrated for its ecosystem approach to managing krill and for setting precautionary catch limits [62,122]. The CCAMLR has also implemented fishery closures of many stocks, which were depleted fisheries in the region prior to the CAMLR Convention’s entry into force [61,62,92]. The CCAMLR has arguably been successful at many aspects of ensuring sustainable, legal, and ecosystem-based harvest, thus the temporal trend is for improvement of this Target Element.

Nonetheless, much concern exists that the CCAMLR is now facing greater difficulties than before for several reasons. First, IUU fishing still persists in the CAMLR Convention Area [90]. Furthermore, interest in the Southern Ocean krill (*Euphausia superba)* and toothfish (*Dissostichus eleginoides* and *D. mawsoni*) fisheries is growing, along with pressure to increase catches in the region [63,75,87]. As mentioned above, krill populations are declining in some areas, causing uncertainty about sustainable catch limits and krill vessels are encroaching on foraging areas of penguin populations [89]. The ecosystem impacts of toothfish fishing remain largely unknown [91]. Evidence of ‘fishing down the food web’ has also been provided for parts of the Southern Ocean [123]. In addition, some CCAMLR Member States are now re-interpreting ‘rational use’ as an unequivocal right to fish rather than a mandate for conserving resources and the ecosystem [124]. Fishing states in the CCAMLR now outnumber non-fishing states 5:3, unlike the situation when the Convention was signed (1:2 ratio of fishing to non-fishing states) [33]. Recent work suggests that in the Ross Sea region both fish size and abundance of the targeted Antarctic toothfish (*D. mawsoni*) declined in McMurdo Sound between 1972 and 2011 [125]. Subsequent work has revealed, in the latter case, however, that catch rate, fish size and fish age have returned to 2002 levels [126], highlighting the need for better long-term information. Indeed, such information has long been a challenge to the CCAMLR, though much work is done by its Scientific Committee and Working Groups to provide the evidence base for decisions.

Based on the evidence available, there is progress toward reaching this Target Element, though given the challenges facing the CCAMLR (summarized most recently in Ref. 75) it is unlikely to be reached by 2020.

*6.2 Recovery plans and measures are in place for all depleted species*

Under the CAMLR Convention rules, the Commission can close fisheries to enable species to recover, and has exercised this option in the case of overexploited populations [38,127]. When the CAMLR Convention first entered into force in 1982, many fish stocks particularly around sub-Antarctic islands, had previously been fished to the point of commercial extinction. In accordance with their precautionary mandate, the CCAMLR immediately closed these areas when the Convention came into force. However, despite being closed for more than three decades, many of these species have yet to recover (e.g., *Notothenia rossii)* and young life history stages continue to be caught as bycatch in ongoing krill fisheries [128]. Furthermore, due to high levels of IUU fishing, many toothfish stocks were heavily depleted. While the CCAMLR closed depleted stocks to fishing (e.g., on Ob and Lena banks), many stocks have yet to demonstrate recovery [127]. These slow recoveries illustrate the overharvesting vulnerability of Antarctic fishes, as previously noted [92]. Despite recommendations of the CCAMLR performance review [43] that recovery plans should be developed for depleted stocks, and scientific work undertaken providing the basis for doing so, no recovery plans are in place for depleted stocks in any of the CCAMLR’s management areas. In consequence, no progress towards or retreat from this Target Element by 2020 has been made.

*6.3 Fisheries have no significant adverse impacts on threatened species and vulnerable ecosystems*

Much progress has been made by the CCAMLR in reducing the extent of bycatch of threatened procellariiform (mostly albatross) species originally substantially affected by toothfish longline fishing in the Southern Ocean [129-135]. Through Conservation Measures based on a range of scientific evidence [121,136,137], incidental seabird mortality through licensed toothfish fishing is now minimal [138]. However, some mortality from IUU fishing is still likely, and IUU fishing has not been entirely eliminated from the region [90], with trade malpractices, such as mislabelling, enabling the laundering of IUU catches [139]. Importantly, Southern Ocean procellariforms also forage in waters beyond CCAMLR jurisdiction. Thus, mortality due to incidental bycatch from Southern Ocean fishing continues to be a problem for threatened species, with many populations declining [140], although measures are also being put in place in areas adjacent to the CAMLR Convention Area to reduce mortality [141].

Occasionally Southern Ocean fisheries also cause incidental mortality of threatened marine mammals. Toothfish fisheries report frequent interaction and depredation by killer whales and sperm whales, and less frequently by other mammals (e.g., Antarctic minke whale). Mortality of marine mammals (including killer whales, sperm whales, minke whales, and elephant seals) is occasionally reported throughout the CAMLR Convention Area, though interactions remain difficult to quantify [142,143]. Trawling operations for icefish (*Champsocephalus gunnari)* and toothfish occasionally cause mortality in mammals [144]. In the past, krill operations frequently caused mortality among mammals (e.g., 95 Antarctic fur seals killed in the 2005/06 season [144]). Since 2008, the CCAMLR has required marine mammal exclusion devices in all krill fisheries and incidental catches have subsequently diminished [38].

CAMLR Conservation Measures have also been effective at minimizing impacts on VMEs based on rules to ensure that when VMEs are identified, they are then protected by fishing prohibitions [36-38,145,146]. However, the location of VMEs in the whole of the Southern Ocean is largely unknown. Often VMEs are accidentally discovered by fishing operations, and much remains to be done to secure the efficacy of the Conservation Measures [75]. MPAs provide an opportunity for true ecosystem-based management as mandated by the CAMLR Convention [69], and progress is being made towards comprehensive establishment (see Target 11 below; [32]). In consequence, while progress is being made towards this Target Element, it is unlikely to be reached by 2020.

*6.4 The impacts of fisheries on stocks, species and ecosystems are within safe ecological limits, i.e. overfishing avoided*

The difficulty of quantifying safe ecological limits [13,14] along with the evidence presented for the previous target elements suggests that neither progress toward or retreat from this Target Element by 2020 is being made, but with relatively low confidence in this assessment.

**Target 7 Areas under agriculture, aquaculture and forestry managed sustainably, ensuring conservation of biodiversity**

This Target was not addressed because these activities are currently not undertaken on the Antarctic continent or in the Southern Ocean. Krill products are used in the aquaculture industry [63], but the sustainability of fish and invertebrate stocks falls within Target 6.

**Target 8 Pollution, including from excess nutrients, has been brought to levels that are not detrimental to ecosystem function and biodiversity**

*8.1 Pollutants (of all types) have been brought to levels that are not detrimental to ecosystem function and biodiversity*

A variety of pollutants, including micropollutants, enter the Antarctic environment either locally through past or current station and shipping practices, or from distant sources by abiotic (long range atmospheric or oceanic transport) or biotic (e.g., seabirds visiting other continental areas) means [147-150].

Pollutants entering the Antarctic from external locations encompass a wide range and include well-known (e.g., heavy metals; persistent organic pollutants (POPs)) and emerging pollutants (e.g., flame retardants), as well as plastic debris [148,150,151-154]. Some of these pollutants have pronounced physiological and/or demographic effects on species in the region [147,155]. A notable recent study has demonstrated substantial negative demographic consequences of heavy metals and POPs for a Wandering Albatross population which typically forages in the CAMLR Convention Area [156]. Moreover, plastic debris may hold especially high risks for Southern Ocean seabirds and is expected to increase in impact into the future [157]. The situation for microplastics remains relatively poorly known, but one recent study has shown that between the East Antarctic and Hobart, Australia, the concentration of microplastics is lower than elsewhere, but still high enough to indicate a global distribution of marine plastic pollution [158].

Ice core records demonstrate that lead pollution on the continent increased from the 1880s, with peaks in the 1900s associated with a single source in Australia, though both flux and concentration have declined since the 1990s [159]. Similarly, the POPs hexachlorobenzene and hexachloroyclohexanes have declined over the last 20 years in the region (see Ref. 160 for a review of POPs in the Antarctic environment). Trends for change in pollutants from distant sources and their regulation are largely determined by global measures and their efficacy. These include changes in ozone depleting substances that have a marked effect on stratospheric ozone above the continent [161]. Hence, from the perspective of long range transport to Antarctica and the Southern Ocean, trends for this Target Element are in keeping with those globally [11], although pollutant levels on the continent and in the Southern Ocean are typically much lower overall than those elsewhere [147,148,150,153,158,162; but see Ref. 157). Nonetheless, remobilisation of legacy contamination through glacial ablation or melt [163] may be particularly problematic as melt increases over many areas of the continent, a trend forecast to continue [164-167].

Local pollution from scientific stations has been substantial in the past [97,147,148,168] and continues [29,169-171], including flame retardants [172], personal care products [173], and microbial contamination [174,175]. Much information has demonstrated the presence of and/or negative effects of this pollution at the genomic, physiological and higher levels of organization of Antarctic biodiversity [147,176-182].

Since the entry into force of the Environmental Protocol in 1998, and especially given the provisions of Annex III on waste management and Annex IV on marine pollution, much effort has now been made to reduce local sources of pollution and clean up previously contaminated sites [183], except where these clean-up practices may worsen the situation (see discussion in Refs. 184, 185). Although much is being done to reduce local sources of pollution, these are still not fully under control [100], and information suggests that activities previously thought to have minimal pollution impact, such as tourism, may have larger than anticipated consequences [186]. Overall, the situation has improved steadily on the continent since the initial establishment of many stations during the International Geophysical Year (1957-1958) [187,188], though indications are that levels will not be reduced to the extent that these are not detrimental to biodiversity and ecosystem functioning. Thus, while progress is being made towards reaching this Target Element, it is unlikely to be reached entirely by 2020.

*8.2 Pollution from excess nutrients has been brought to levels that are not detrimental to ecosystem function and biodiversity*

Pollution from excess human-derived nutrients was not considered here given the low level of activity likely to introduce excess nutrients in the region. Most Antarctic stations (> 80%) [96] are coastal and discharge their human waste into the sea, introducing local nutrient pollution. This is also the case for fishing, national operator and tourist vessels. The contribution is thought to be overwhelmed at many sites by the faecal material production by wildlife [189], although changes to local systems can take place, including as a consequence of release of microbial contamination [97,189]. Many stations are now changing sewage treatment practises, achieving standards better than national requirements for water quality [97].

Many terrestrial Antarctic environments, removed from the coast and free of seabird colonies, are depauperate in nutrients, including carbon [190]. Thus, hydrocarbon spills have the potential to act as nutrient pollutants. Substantial regulations for hydrocarbon spills and advice for clean-up are in place via the CEP [183], and technologies have been a significant topic of research [191], though hydrocarbon spills continue to be recorded [103].

**Target 9 Invasive alien species and pathways identified, species controlled, and pathways managed**

*9.1 Invasive alien species identified and prioritized*

Much research has been undertaken on this topic for the broader Antarctic region. The most significant terrestrial invasive alien species have been identified both for the sub-Antarctic islands and the continent [12,79,192,193]. Species introduced to and established on the continent are regularly documented in reports to the CEP, with indications of which species are most significant [193]. Several reports have now been made of marine invasive alien species being captured south of 60°S [97,194], although confirmation of actual establishment is not available. Information on introduced diseases is also growing [195,196]. The potential risks from invasive alien species have also been highlighted and have been raised in the CEP (see discussion and synopsis in Ref. 197), though no further action has been taken in the marine context, apart from work on regulation of pathways (see below). Nonetheless, the CEP and ATCM, along with ATCPs, have made non-native species a priority and a careful watch is kept on this area. While formal prioritization is yet to be undertaken, the underlying data required to do so are available and proposals for how this can be done have been made [79,194,197-199]. On this basis, the Target Element will be achieved by 2020.

*9.2 Pathways identified and prioritized*

The pathways for alien and invasive alien species entry into the Antarctic region are now well understood from the microbial level to all plants and animals [79,97,174,194,196,200-207]. These include marine and terrestrial pathways, most of which are subject to control [193,208,209]. Attention has also been given to intra-regional pathways, because of substantial dissimilarity among Antarctica’s Conservation Biogeographic Regions, and significant local genetic structuring [3,39]. Pathway management interventions include gear cleaning and inspection, educational material to inform all visitors to the region [193,204,210], and provisions for ballast water exchange [208]. Intra-regional movement interventions are also the subject of a SCAR Code of Conduct for terrestrial field research in Antarctica [211]. Hull fouling is well understood as a pathway and has been prioritized for further consideration, especially given the influence of sea ice on hull fouling and changing sea ice conditions [200-202,207]. This Target has already been met and in many ways exceeded given the substantial actions in place to restrict pathway efficacy.

*9.3 Priority species controlled or eradicated*

Eradication of invasive alien species has been undertaken on the Antarctic continent [193], reflecting considerable attention to the matter in the sub-Antarctic [192,212]. In the case of eradication, priority species such as the grasses *Poa annua* and *Poa pratensis* have been removed, or plans are in place to attempt to remove them [12,193,197,213]. Nonetheless, some priority species at some locations have yet to have plans set in motion for control or eradication. For groups such as terrestrial invertebrates, which are increasingly being recorded from the Treaty region and perhaps underestimated in their extent and significance, control or eradication may prove problematic or impossible [197,198]; see also discussion in Ref. 214). Little attention has been given to control or eradication of marine alien species. Thus, while much is being done in this area the Target Element is unlikely to be fully met by 2020.

*9.4 Introduction and establishment of IAS prevented*

New alien species establishments continue to be recorded both in terrestrial and marine systems [12,104,192,194]. Moreover, it is expected that with rapidly changing climates, and increasing human activity, especially along the Antarctic Peninsula, establishment barriers will be lowered [12,47,79,199]. Many terrestrial protected areas are also highly susceptible to invasion both because of their location [94], and the management arrangements in place for them, which do not preclude introductions to immediately adjacent land in the same ice-free area [39]. Implementation of arrangements to prevent introduction of IAS are also not consistent among those operating in the region [197]. Thus, this Target is unlikely to be fully achieved by 2020 given the changing conditions, despite the high priority given to the non-native species problem by the CEP [209]. In part to help address these problems, an indicator for biological invasion for the region has been developed, which includes state, pressure, drivers and responses [12]. Particularly susceptible sites have been identified [12], and procedures for surveillance and responses to new colonisations have been developed [39,197,198]. What is now required is take-up of these new developments by the ATS to improve the likelihood of meeting this Target Element.

**Target 10 Anthropogenic pressures minimized on ecosystems vulnerable to climate change and ocean acidification**

Southern Ocean ecosystems are thought to be especially vulnerable to ocean acidification as a consequence of the physical conditions of the Southern Ocean, leading to an expectation of widespread aragonite undersaturation by 2050 [44,215]. Several studies have argued that these effects are already being felt by marine organisms, or will rapidly become significant [216,217], though with much subsequent debate about the matter [218,219], and indications that some species may not be as sensitive [220]. Ecosystem impacts of climate change and fishing also seem likely to be significant [44,215,221]. Much of the policy change required to give effect to minimizing anthropogenic pressures on the Southern Ocean ecosystem lies outside of the direct jurisdiction of the ATS, though within the means of many ATCPs to give effect to through their participation in other international agreements. Despite policy improvements, CO_2_ emissions appear to be tracking the highest estimates of the IPCC representative concentration pathway scenarios [222], indicating that, if anything, for the Southern Ocean, the situation is worsening. Nevertheless, because of minimal local influences, we elected not to assess this Target.

**Strategic Goal C. Improve the status of biodiversity by safeguarding ecosystems, species and genetic diversity**

**Target 11 Conservation through ecologically representative protected areas**

*11.1 At least 17 percent of terrestrial areas and inland waters within systems of protected areas or other effective area-based conservation mechanisms*

Because Article 2 of the Environmental Protocol designates Antarctica as a ‘natural reserve’, the entire continent is often assumed to have a high level of protection, consistent with an IUCN-designated protected area (PA) [223]. By contrast, the IUCN itself does not consider the continent a PA [224], no overall strategy exists for managing the continent as a PA, and the Environmental Protocol provides a mechanism for designating Antarctic Specially Protected Areas (ASPAs). In consequence, the continent as a whole cannot be considered a PA.

Evidence for this Target Element is well developed. Although Antarctica’s ice-free area is *ca*. 45 886 km^2^ (compared with a total area of approximately 14 million km^2^), as of 2016 < 4% (1628 km^2^) of this area is formally designated within ASPAs for the purpose of biodiversity conservation (S1 Table). Since 2014, new ASPAs have been designated and some have been de-designated [95], but the rate of designation is low with no new Antarctic Conservation Biogeographic Regions (ACBRs) included in ASPAs since 2014. In consequence, this Target Element is unlikely to be met by 2020.

*11.2 At least ten percent of coastal and marine areas within systems of protected areas or other effective area-based conservation mechanisms*

Within the Antarctic Treaty area, south of 60°S, a considerable diversity of marine habitats exists, identified through detailed, spatially explicit analyses [225-227]. They include unusual and vulnerable systems such as deep-sea hydrothermal vents [228], and support many taxa, often equalling the diversity of tropical non-reef ecosystems [114]. Moreover, much evidence exists for increasing fishing interest and impacts in the region [123,124], which may be compounding the impacts of climate change.

In 2002, in an effort to meet global MPA targets and in recognition of the value of MPAs as a biodiversity conservation and fisheries management tool, particularly in the face of climate change, the CCAMLR committed to designating a network of MPAs in the Southern Ocean [33]. Initial progress was slow, with a single ~94,000 km^2^ MPA designated by 2009, south of the South Orkney Islands [229]. In 2016, an additional ~1.55 million km^2^ was declared a Marine Protected Area in the Ross Sea, to come into force in December 2017 [32].

States have also made progress in designating MPAs around many sub-Antarctic islands north of 60°S, which fall both within the CAMLR Convention Area and are under national jurisdiction (as designated prior to the signing of the CAMLR Convention). These include the Heard Island and McDonald Islands (HIMI) marine reserve (~71,000 km^2^; governed by Australia [230]) and the Prince Edward Islands MPA (~180,000 km^2^; governed by South Africa [231,232]) which extends north of the CAMLR Convention Area boundary. The United Kingdom has also designated an MPA around the South Georgia and South Sandwich Islands (~1.07 million km^2^) [233], although this MPA, and sovereignty of the islands, remain contested by Argentina. Combining the total area protected from these subantarctic MPAs plus the South Orkney Islands and Ross Sea MPAs, and several marine ASPAs (see S1 Table) *ca.* 8% of the total CAMLR Convention Area (35,716,100 km^2^) will be protected as of 2018. An East Antarctic MPA has also been the subject of discussion [34,225,234,235], which if declared by 2020 would mean that this Target Element would come close to being met (ca. 9.5% of the CAMLR Convention Area would be under protection). Further planning is underway for MPAs in the Weddell Sea and Antarctic Peninsula areas, but they have yet to come under formal negotiation [90]. It remains unclear whether or not the CCAMLR will meet this Target Element by 2020, given the time taken to reach agreement over the Ross Sea MPA [236], and substantial fisheries interests especially in the Antarctic Peninsula region [34].

*11.3 Areas of particular importance for biodiversity and ecosystem services conserved*

Article 3.2 of Annex V to the Environmental Protocol states that Parties shall seek to identify areas with important or unusual assemblages of species, including major colonies of breeding native birds or mammals, and the type locality or only known habitat of any species. The substance of this Target Element is thus addressed by the Environmental Protocol. Progress is also being made in the conservation of areas of particular importance for biodiversity. For example, unusual concentrations of wildlife or unusual combinations or numbers of species are frequently cited as reasons for ASPA designation (26 of 72, see also Ref. 94). An invertebrate example comes from Marion Nunataks, Charcot Island, where no mite predators or Collembola have been recorded [237], and the site is now included in an ASPA (ASPA No. 170 [238]). Recent analysis has identified 204 terrestrial Important Bird Areas (IBAs) in Antarctica [239]. Elsewhere, IBAs have been treated as ‘*areas of particular importance for biodiversity’* in the language of Aichi Target 11 in global and regional assessments [9, 240], and their coverage by protected areas is used to report progress against the Sustainable Development Goals [241]. Several of these are already included in ASPAs, but the ATCPs have also resolved that the CEP should advise on the extent to which these IBAs are, or should be, represented in the ASPA system [242]. By contrast, other unusual sites, such as one in Ellsworth Land that is dominated by tardigrades and free of nematodes [243], have not been afforded ASPA status. In addition, no systematic attempt has yet been made to protect type localities.

For marine systems, much discussion has taken place within the CCAMLR about protecting VMEs [37,144], typically defined as benthic assemblages susceptible to anthropogenic disturbance. The CCAMLR Secretariat maintains a register of VMEs [36] and several conservation measures are in place that restrict disturbance to VMEs [38], though the area they protect is far from comprehensive and their efficacy is yet to be fully established [75]. In an effort to guide MPA establishment, CCAMLR scientists and other experts conducted extensive Southern Ocean bioregionalisation, identifying areas of critical importance for biodiversity and the ecosystem [225,226]. Many of these are not yet protected.

Overall, the evidence suggests that although progress is being made on protecting areas of particular importance for biodiversity, the Target Element is unlikely to be met by 2020.

*11.4 Protected areas are ecologically representative*

Article 3.2 of Annex V to the Environmental Protocol states that: ‘Parties shall seek to identify, within a systematic environmental-geographic framework, and to include in the series of Antarctic Specially Protected Areas…representative examples of major terrestrial, including glacial and aquatic, ecosystems…’. Thus, the substance of Target 11 is included within the Environmental Protocol. Much progress has also been made to identify what might be considered the systematic environmental-geographic framework on which to base the identification of potential ASPAs [3,95,244]. And this work has found its way into formal recommendations by the ATCPs [245,246].

Nonetheless, a recent analysis has demonstrated that of the 15 ACBRs, then recognized for ice-free Antarctica, no ACBR has more than 10% of its area included within ASPAs, and five have no ASPAs at all for the purposes of conserving biodiversity [94]. Just two of the ACBRs contain most ASPAs, representing 74% of all ice-free ASPAs designated for the protection of biodiversity.

Refining the taxonomic scope of the assessment to the macroscopic terrestrial flora, reveals a similar picture [56]. The 33 ASPAs that contain terrestrial macroscopic vegetation fail to represent vegetation present in six ACBRs, with a further six having <0.4% of their area within an ASPA designated for the protection of botanical values. Across the continent, protected vegetation cover amounts to 16.1 km^2^, with more than 50% of that in a single ASPA, and over 96% of it contained in just two of the 15 ACBRs that were considered in that analysis.

Four ASPAs (ASPA 119 Davis Valley and Forlidas Pond, Dufek Massif, Pensacola Mountains; ASPA 131 Canada Glacier, Lake Fryxell, Taylor Valley; ASPA 147 Ablation Valley and Ganymede Heights, Alexander Island; ASPA 172 Lower Taylor Glacier and Blood Falls) are concerned with inland waters, despite their extraordinary diversity on the continent [247,248]. Concerns about the efficacy of protection for the microbiota, uniquely important in an Antarctic terrestrial context, have also been raised [175].

In consequence, no substantive progress has been made in designating ASPAs according to the information now widely available, leaving the continent one of the least protected terrestrial environments on Earth (see also Ref. 249). Thus, from a terrestrial perspective, neither progress towards or retreat from this Target Element is being made.

Across the Southern Ocean, 23 benthic ecoregions and 20 pelagic bioregional clusters have been identified [226,250]. Of these, 11 benthic ecoregions and 13 pelagic bioregions are represented in the five Southern Ocean MPAs discussed previously (South Orkneys, South Georgia, HIMI, Ross Sea, PEI). MPA proposals in the East Antarctic and Weddell Sea currently being negotiated by the CCAMLR would cover an additional 9 benthic and 2 pelagic ecoregions. Thus, progress is being made towards ecological representation, though complete coverage is unlikely to be achieved by 2020.

*11.5 Protected areas are effectively and equitably managed*

Quantitative analyses have demonstrated that the terrestrial ASPA network is at high risk of non-indigenous species establishment, and located closer to sites of tourist landing and scientific activity than expected when compared to the same number of randomly selected ice-free sites [94]. Moreover, evidence shows that establishment of an ASPA is no guarantee of compliance with management provisions aimed at reducing environmental degradation [78,96,103]. Such provisions have also not considered standard biological principles, such as dispersal and connectivity, which may elevate risk [39]. In consequence, while management plans have to be developed for the designation of any ASPA, and revised every five years, these plans are not thought to be fully effective [39,96,251], with little evidence to suggest that steps are being taken to address the situation.

For the four existing CAMLR Convention Area MPAs (i.e. excluding the Ross Sea MPA which has not entered into force), there is limited evidence available to determine whether they are being effectively managed. The HIMI marine reserve, adopted in 2002, appears to be meeting many of its management goals, but this is largely due to passive protection via the area’s isolation and harsh conditions rather than active management [252]. Limited information is available to determine effective management of the Prince Edwards Islands MPA, which was only adopted in 2013 [253]. The 2009 adopted South Orkney Islands MPA did not have a research and monitoring plan until 2014, and has made limited progress towards its MPA goals [254]. The South Georgia and South Sandwich Islands MPA was adopted in 2012 and has a management plan [233], but insufficient time has elapsed to assess its effectiveness and the MPA has been criticized as being inadequate for, among other matters, having minimal no-take zones [255].

In consequence, progress is not being made toward meeting this Target Element by 2020, nor is there retreat away from it, though confidence in this assessment is low.

*11.6 Protected areas are well connected and integrated into the wider landscape and seascape*

Owing to the large geographic extent of Antarctica and the Southern Ocean, the isolated nature of Antarctic terrestrial ice-free areas and the likelihood of substantial differences among them, along with similar patterns of differential connectivity and isolation in marine areas [115], connectedness among protected areas in the region was not considered relevant. Indeed, for terrestrial areas the converse is perhaps of more importance [3,39].

Much scientific work has been undertaken on developing an integrated protected area network for the region [3,94,95,225-227], and understanding of connectivity in marine and terrestrial systems is developing [114,117,256]. While the ATCPs have responded to this work by recognizing its value in the context of developing protected areas [242,245,246], little further work has been undertaken to integrate protected areas. In consequence, while progress is being made towards this Target Element, it is unlikely to be reached by 2020.

**Target 12 Extinction of known threatened species prevented and status improved**

*12.1 Extinction of known threatened species has been prevented*

Antarctica is, remarkably, the only continent from which an anthropogenic extinction has yet to be recorded [257-259]. Holocene avian extinctions have, by contrast, taken place on some sub-Antarctic islands [258], with several local extirpations also known [260]. No extinctions are known from the Southern Ocean either, though substantial declines of commercially significant fish species and of whales, seals and penguins were associated with exploitation commencing in the 19^th^ Century [61,261,262]. Some trends and analyses have suggested that current commercially exploited fish species in the Southern Ocean, particularly toothfish, are on the decline, though in some cases the data are insufficient to demonstrate this with confidence [123,125,126]. For some whales and seals, populations have recovered, although comprehensive assessments for cetaceans are not available (see discussion below in Target Element 12.2). Several threatened bird species on the IUCN Red List of Threatened Species (hereafter IUCN Red List [263]) continue to decline ([264]; see also S2 Table). In consequence, although information is limited, it appears that this Target Element may be met by 2020, but with significant departures after that time if population declines and climate change and invasive species impacts continue [12,215,265,266].

*12.2 The conservation status of those species most in decline has been improved and sustained*

This Target Element was not assessed explicitly because so little work has been done on status assessments for the region. No assessments of the conservation status of Antarctic terrestrial species have been undertaken (though for terrestrial sub-Antarctic birds such as *Anas eatoni* (Vulnerable) and *Chionis minor* (Least Concern) this has been done), with no terrestrial biota from the continent represented on the IUCN Red List of Threatened Species [263] other than birds that breed in Antarctica.

All birds and mammals, and some fishes, which occur in the region south of 60°S, have been assessed for the IUCN Red List, and these are mostly species which breed on the continent (or Southern Ocean islands) and forage in the CAMLR Convention Area. The red list status of many of the seabird species is one of decline ([12,264]; S2 Table), as a consequence of incidental mortality from fishing and from the impacts of invasive alien species on the islands at which they breed. Considerable effort is now being expended to eradicate these alien species on key breeding sites [212,267]. Fisheries management strategies have largely mitigated seabird by-catch [138], however incidental catches from IUU and in fisheries outside the CCAMLR area remains a concern [264].

Further south, invasive alien species are not of concern for seabirds. Here, climate change is having substantial impacts on species breeding on the continent, such as Adélie and Chinstrap penguins [266,268]. Although climate change may also be an important factor influencing Emperor penguins [269], other evidence suggests that these assessments require further consideration [256]. Fishing impacts, notably for krill, are thought to constitute a further threat to several seabird species, such as penguins [265]. These impacts have been more broadly recognized, including by the ATCPs [270,271] and by the CCAMLR [75]. While formal conservation measures have yet to be implemented, attention given to the matter indicates that progress is being made towards reaching this Target Element, though climate change impacts and increased interest in fishing [124] suggest that it is unlikely to be met by 2020.

By contrast with the situation for birds, that for seals and whales is typically improving as a consequence of the cessation of sealing and whaling in the Antarctic [117,261,262,272-277], although with several significant exceptions. For several species, modern abundance data are not available to evaluate trends. Nonetheless, as for other Antarctic species, the impacts of ocean acidification and climate change are likely to be substantial over the longer term [44,215]. In consequence, while progress is being made toward Target 12, much remains to be done, suggesting that it is unlikely to be met by 2020.

**Target 13 Genetic diversity of cultivated, farmed and culturally valuable species maintained**

Farming is not typically undertaken on the Antarctic continent. Limited hydroponic facilities provide some cultivation of fresh produce at several stations [12], and guidelines for such facilities have been developed and reported on at the CEP [278]. Species such as Antarctic fur seals and some whales could be considered culturally valuable. In the case of the former, populations have recovered since their substantial decline in the previous centuries, and genetic diversity remains high [117,273,274]. Information on genetic diversity of Antarctic whales is only now becoming widely available [e.g., 279,280]. In consequence, this Target was not assessed.

**Strategic Goal D. Enhance the benefits to all from biodiversity and ecosystem services**

**Target 14 Ecosystem services safeguarded**

*14.1 Ecosystems that provide essential services, including services related to water, and contribute to health, livelihoods and well-being, are restored and safeguarded*

The Southern Ocean and the Antarctic ice sheet and shelves are the greatest source of ecosystem services in the region. These services include carbon sequestration and the maintenance of sea levels, and to a somewhat lesser extent the provision of ocean fishery resources, though Antarctic krill has the potential to comprise 11% of global marine fisheries landings, and with a variety of other services [281]. Much evidence now exists to indicate that ecosystem services from the Southern Ocean will be substantially affected by ongoing Greenhouse Gas (GHG) emissions [164,282,283]. Although steps are being taken globally to reduce GHG emissions, indications are that the rate of increase in atmospheric CO_2_ concentrations is on track for the highest of the IPCC’s representative concentration pathways [222]. In consequence, ecosystem service delivery from the Southern Ocean and from Antarctic ice mass is likely to decline, resulting in potentially substantial consequences for carbon sequestration (see discussions in Refs. 284,285) and sea level rise [164]. The consequences of ocean acidification are also likely to become more significant, given the susceptibility of the Southern Ocean and its biota [44]. Thus, as a consequence of factors that lie outside the control of the ATS, but not that of the ATCPs in their roles also as State Parties to other agreements, the overall trend is away from this Target.

**Target 15 Ecosystem resilience and the contribution of biodiversity to carbon stocks have been enhanced, through conservation and restoration, including restoration of at least 15 per cent of degraded ecosystems, thereby contributing to climate change mitigation and adaptation and to combating desertification**

Target 15 was considered as part of Target 14 because restoration is not a major activity in the region, despite discussion of environmental repair and remediation at the CEP [105]. Such remediation and repair typically has to do with degradation owing to previous pollution [183,286-288]. Desertification is not a concern owing to the typically dry physical environment of the continent [289].

**Target 16 Access to genetic resources and benefit sharing**

*16.1 The Nagoya Protocol is in force*

This target was not considered owing to the governance arrangements for the Antarctic Treaty area (south of 60°S) [25,26].

*16.2 The Nagoya Protocol is operational, consistent with national legislation*

Here we considered access and benefit sharing in the context of bioprospecting in the Antarctic. Bioprospecting has long been a concern of the ATCPs, with discussion of the matter arising at several of the ATCMs [290-292]. Many patents have been registered for products derived from the region’s biodiversity [293,294], several works have considered the matter [295-299], and a review has provided an update of the current status of bioprospecting in the region [300]. Despite consideration of bioprospecting [290-292], including through invitation to SCAR to review the matter [301], and most recently through Resolution 6 of 2013 [302], the ATCPs have not yet considered any arrangements to give effect to access and benefit sharing [295,299,300]. Rather, regulation of bioprospecting has not been considered in detail, although several ATCPs have presented papers to Treaty Meetings in an attempt to move the discussion forward [291]. Since 2013, bioprospecting has been raised occasionally at the ATCMs, but with little substantive discussion or progress. In consequence neither progress toward nor retreat from the Target Element by 2020 is being made.

**Strategic Goal E. Enhance implementation through participatory planning, knowledge management and capacity-building**

**Target 17 Biodiversity Strategy and Action Plan**

*17.1 Biodiversity Strategy and Action Plan*

No equivalent plan currently exists for Antarctica and the Southern Ocean. The Environmental Protocol and its Annexes provide an indication of what elements might be included in such a strategy and action plan. The Environmental Protocol has also produced a range of guidelines which provide implementation advice. These include, for example, a non-native species manual [209], guidelines for the operation of aircraft [303,304], a clean-up manual to address environmental risks posed by waste disposal sites or other contaminated areas [183], and a guide to the preparation of management plans for APSAs [305]. The CEP also considers its priorities annually, through its five-year rolling work plan and adjusts this to reflect its current concerns [306].

The CCAMLR likewise has a range of conservation measures that reflect elements of this target [1,38,75]. The 2008 CCAMLR Performance Review noted, however, ‘…that there needs to be the development of an overall strategy for protection of biodiversity…’ [43], which has yet to be done. Importantly, the guidelines and priorities of the CEP and the CCAMLR are not aligned formally such as might be done under the umbrella of an Antarctic biodiversity strategy and action plan.

SCAR and partners are in the process of concluding a document on Antarctic Conservation in the 21^st^ Century, which could form the basis for an Antarctic biodiversity strategy and action plan. To have any effect however, such a plan would have to be endorsed by the ATCPs and by CCAMLR Member States. Within the timeframe set by Target 17 this could be achieved, but would take substantial collaborative effort, and is unlikely to be done by 2020.

*17.2 Antarctica included in National Biodiversity Strategy and Action Plans*

Many of the 29 Consultative and 24 Non-Consultative Parties to the Antarctic Treaty, and the 24 State Members of the CCAMLR, have developed National Biodiversity Strategy and Action Plans (NBSAPs) in accordance with the CBD guidelines (with exceptions such as the USA, which has signed, but not ratified the CBD). Of 53 Treaty Parties, 51 have NBSAPs (excluding USA, and Monaco’s is still under development), but only eight of these mention the Antarctic region, and three (Belgium, Japan and New Zealand) devote some discussion to the region (S3 Table). Among the states making claims to Antarctic territory (recognizing the effect of Article IV of the Antarctic Treaty on these claims) – viz. Argentina, Australia, Chile, France, New Zealand, Norway, United Kingdom – either no statement is made about Antarctica in their NBSPAs (Chile, U.K.) or the extent of discussion is no more extensive than that of most other states. Thus, Antarctica and the Southern Ocean are unlikely to be included in NBSAPs via this route by 2020.

**Target 18 Indigenous Knowledge**

Although some nations (Argentina, Chile) have registered births in Antarctica, the continent has no indigenous population. This Target was therefore not considered.

**Target 19 Knowledge Base Improved and Knowledge Transferred**

*19.1 Knowledge, the science base and technologies relating to biodiversity, its values, functioning, status and trends, and the consequences of its loss, are improved*

Science, including investigations of biodiversity both in the Southern Ocean and on continental Antarctica, is supported by a range of nations. Indeed, a substantial scientific presence in the region is a pre-requisite for Consultative Party status within the Antarctic Treaty. One recent estimate has science-related spending at *ca*. US$ 950 million for the region in 2012 [4], and suggests that spending likely exceeds US$ 1 Billion per year. What proportion of this is devoted to biodiversity science is difficult to determine. Nonetheless, the considerable extent of biodiversity research is reflected by the large representation of such work at the SCAR biennial Open Science Conferences, and in the various reports of the SCAR Scientific Research Programs and Standing Scientific Groups [307].

No formal mechanism for tracking biodiversity-related activity in the region is available. In the most recent SCAR Scientific Horizon Scan [57,58], 29 of the 80 most pressing questions were concerned with biodiversity, suggesting that substantial extra investment is required to understand and improve the status of biodiversity in the region. Thus, although progress is being made, this Target Element is unlikely to be met by 2020.

*19.2 Biodiversity knowledge, the science base and technologies are widely shared and transferred and applied*

The primary mechanisms for biodiversity knowledge transfer in the region are via national submissions to the CEP and to the CCAMLR and its subsidiary science bodies. In the case of the CEP, the ATCPs recently recommended, by Resolution [308], that an environmental information sharing mechanism, the Antarctic Environments Portal [309], be considered a mechanism for making high quality, timely scientific advice available for use by the members of the CEP and the ATCPs. The Environments Portal provides a range of content on environmental matters of direct interest to the Treaty Parties (e.g., wildlife diseases, non-native species, climate change impacts on penguins). In the case of the CCAMLR, biodiversity knowledge sharing would occur largely via national submissions at the science working group level, which may transfer up through to the CCAMLR’s Scientific Committee and to the Commission itself. Through the CCAMLR’s efforts towards MPA planning over the last decade, biodiversity information has been widely gathered and shared between the CCAMLR’s science bodies. In some cases, this knowledge has been shared beyond the CCAMLR, to the public, for example via published bioregionalisation documents [225]. Thus, mechanisms for transferring knowledge to decision-makers and the public have improved, given these recent developments.

Nonetheless, much evidence exists that this knowledge has yet to be fully taken up or that action has increased to do so. For example, a recent analysis has demonstrated that despite the availability of site-specific population size and trend data for species (e.g., in Ref. 310) that are the focus of given ASPAs, ASPA management plans fail to include these data [251]. Likewise, despite recent demonstrations that terrestrial protected areas fail fully to represent the Antarctic Conservation Biogeographic Regions [94], no proposals have yet been made to change the situation. Likewise, despite the gathering of extensive data sets on biodiversity to form the basis of MPAs in the Southern Ocean, CCAMLR States have been slow to declare representative MPAs [34]. In consequence, this Target element is unlikely to be met by 2020.

**Target 20 Mobilization of financial resources**

Investment by Parties in Antarctic governance is relatively modest compared to other international arrangements. Member contributions to the annual budget of the Buenos Aires-based Antarctic Treaty Secretariat, which supports the ATCM and CEP, are collectively just less than US$1.4M [311]. Member contributions to the CCAMLR’s general fund amount to approximately US$3.2M [90]. Member contributions to the general fund of the Convention on Biological Diversity are approximately US$13.2M [312]. While the CCAMLR’s Scientific Committee can request funds for specific activities (e.g., organising intersessional workshops or to support peer review of stock assessments), the CEP has no such access to funding.

Nonetheless, despite the absence of an Antarctic biodiversity strategy and action plan, and the limitations on available resources for conservation action in the region, individual ATCPs have invested in activities to improve the status of biodiversity in the region. Examples include organising and hosting intersessional workshops (e.g., on protected areas [313,314]; non-native species [315]; marine protected areas [316]; joint meetings of CEP and CCAMLR’s Scientific Committee [317], and an ATCM Meeting of Experts on Climate Change [270]), and support from Parties directly to activities such as the Environments Portal (supported largely by New Zealand). One of the most significant investments was for the circum-Antarctic Census of Marine Life which resulted in the Biogeographic Atlas of the Southern Ocean [45]. Non-Consultative Parties also contribute to these endeavours, such as demonstrated by the meeting which produced this document. Likewise, NGOs have played a critical role in mobilizing resources to improve the status of biodiversity in the region. These include Oceanites, which has one of the longest running penguin population monitoring programmes along the Antarctic Peninsula [310], ASOC and their associated environmental NGOs, and IAATO, which implements and provides feedback to the CEP on the utility of site specific visitor guidelines [318]; which in turn are updated regularly by the ATCPs [319] to ensure they continue to provide appropriate environmental protection in the context of tourism. IAATO has also invested in the development and distribution among its operators of additional wildlife watching guidance material aimed at minimising disturbance from tourism activities [320]. Nonetheless, these financial resources are likely to fall short of delivering the kinds of information required to reduce losses to biodiversity in the region, especially given the challenges facing it associated with rapid climate change and ocean acidification [57,58].

**References**

1. Miller D. Sustainable management in the Southern Ocean: CCAMLR science. In: Berkman PA, Lang MA, Walton DWH, editors. Science diplomacy. Antarctica, science, and the governance of international spaces. Washington, DC: Smithsonian Institution; 2011. pp. 103-121.
2. Dingwall PR. Progress in the conservation of the Subantarctic Islands. Gland: IUCN; 1995.
3. Terauds A, Chown SL, Morgan F, Peat FJ, Watts DJ, Keys H, Convey P, Bergstrom DM. Conservation biogeography of the Antarctic. Divers Distrib. 2012; 18: 726-741.
4. Dodds K, Hemmings AD. Polar oceans: sovereignty and the contestation of territorial and resource rights. In: Smith HD, Suárez de Vivero, SJ, Agardy TS, editors. Routledge Handbook of Ocean Resources and Management. London: Routledge; 2015, pp. 576-591.
5. Triggs G. The Antarctic Treaty System: A model of legal creativity and cooperation. In: Berkman PA, Lang MA, Walton DWH, editors. Science diplomacy. Antarctica, science, and the governance of international spaces. Washington, DC: Smithsonian Institution; 2011. pp. 39-49.
6. Burgman M, Carr A, Godden L, Gregory R, McBride M, Flander L, Maguire L. Redefining expertise and improving ecological judgment. Conserv Lett. 2011; 4: 81-87.
7. Martin TG, Burgman MA, Fidler F, Kuhnert PM, Low-Choy S, McBride M, Mengersen K. Eliciting expert knowledge in conservation science. Conserv Biol. 2012; 26: 29-38.
8. Drescher M, Perera AH, Johnson CJ, Buse LJ, Drew CA, Burgman MA. Toward rigorous use of expert knowledge in ecological research. Ecosphere 2013; 4: 83.
9. Secretariat of the Convention on Biological Diversity, Montréal. Global Biodiversity Outlook 4. 2014; Available from: http://[www.cbd.int/GBO4](http://www.cbd.int/GBO4).
10. Mastrandrea MD, Field CB, Stocker TF, Edenhofer O, Ebi KL, Frame DJ, et al. Guidance note for lead authors of the IPCC Fifth Assessment Report on consistent treatment of uncertainties. Intergovernmental Panel on Climate Change. 2010. Available from: <http://www.ipcc.ch2>.
11. Tittensor DP, Walpole M, Hill SLL, Boyce DG, Britten GL, Burgess ND, et al. A mid-term analysis of progress toward international biodiversity targets. Science 2014; 346: 241-244.
12. McGeoch MA, Shaw JD, Terauds A, Lee JE, Chown SL. Monitoring biological invasion across the broader Antarctic: a baseline and indicator framework. Global Environ Change 2015; 32: 108-125.
13. Butchart SHM, Di Marco M, Watson JEM. Formulating smart commitments on biodiversity: lessons from the Aichi Targets. Conserv Lett. 2016; 9: 457-468.
14. Oliver TH. How much biodiversity loss is too much? Science 2016; 353: 220-221.
15. del Acebo Ibáñez E, Costa M. Antarctic environmental problems: attitudes and behaviours of young inhabitants of two Argentine cities (Buenos Aires and San Carlos de Bariloche). Polar Rec. 2010; 46: 257-263.
16. Shabudin AFA, Rahim RA, Nor RM, Ibrahim K. Antarctic values and Malaysia’s involvement in Antarctica: perceptions among young citizens of Malaysia. Polar Rec. 2016; 52: 305-315.
17. Tin T, Bastmeijer K, O’Reilly J, Maher P. Public perception of the Antarctic wilderness: surveys from an educated, environmentally knowledgeable European community. In: Watson A, Murrieta-Saldivar J, McBride B, editors. Science and stewardship to protect and sustain wilderness values. Fort Collins: USDS Forest Service Proceedings RMRS-P-64; 2011. pp. 109-117.
18. Neufeld E, O’Reilly J, Summerson R, Tin T. Valuing Antarctica: emerging views from international studies. In: Tin T, Liggett D, Maher PT, Lamers M, editors. Antarctic futures. Human engagement with the Antarctic environment. Dordrecht: Springer; 2014. pp. 233-252.
19. Young P. The last ocean; 2012. Available from: http://[www.thelastoceanfilm.com/](http://www.thelastoceanfilm.com/).
20. IAATO. Visitor briefings. IAATO, Newport, 2016. Available from: <http://iaato.org/visitors-slide-show>.
21. Meduna V. Antarctica reveals secrets to scientists. Radio New Zealand, Wellington. 2015. Available from: <http://www.radionz.co.nz/national/programmes/ourchangingworld/audio/201759551/antarctica's-surprising-biodiversity>.
22. IAATO. Don’t pack a pest. IAATO, Newport. 2016. Available from: <http://iaato.org/dont-pack-a-pest>.
23. ATCM. Protocol on environmental protection to the Antarctic Treaty. Antarctic Treaty Secretariat, Buenos Aires. 1991. Available from: <http://www.ats.aq/documents/recatt/Att006_e.pdf>.
24. CCAMLR. The Convention on the Conservation of Antarctic Marine Living Resources. CCAMLR Secretariat, Hobart. 1980. Available from: http://[www.ccamlr.org](http://www.ccamlr.org).
25. Berkman PA, Lang MA, Walton DWH, editors. Science diplomacy. Antarctica, science, and the governance of international spaces. Washington, DC: Smithsonian Institution; 2011.
26. Saul B, Stephens T. Antarctica in international law. Oxford: Hart Publishing; 2015.
27. IUCN. A strategy for Antarctic conservation. Gland: IUCN; 1991.
28. Walton DWH. The Scientific Committee on Antarctic Research and the Antarctic Treaty. In: Berkman PA, Lang MA, Walton DWH, editors. Science diplomacy. Antarctica, science, and the governance of international spaces. Washington, DC: Smithsonian Institution; 2011. pp. 75-88.
29. Klein AG, Sweet ST, Kennicutt II MC, Wade TA, Palmer TA, Montagna P. Long-term monitoring of human impacts to the terrestrial environment at McMurdo Station. In: Tin T, Liggett D, Maher PT, Lamers M, editors. Antarctic futures. Human engagement with the Antarctic environment. Dordrecht: Springer; 2014. pp. 213-230.
30. ATCM. Measure 18 of 2015 – ATCM XXXVIII – CEP XVIII. Management Plan for Antarctic Specially Managed Area No. 2 McMurdo Dry Valleys, Southern Victoria Land. Antarctic Treaty Secretariat, Buenos Aires. 2015. Available from: [www.ats.aq](http://www.ats.aq).
31. Kennicutt MC II, Kim YD, Rogan-Finnemore M, Anandakrishnan S, Chown SL, Colwell S, et al. Delivering 21^st^ Century Antarctic and Southern Ocean science. Antarct Sci. 2016; 28: 407-423.
32. CCAMLR. CCAMLR to create the world’s largest Marine Protected Area. CCAMLR Secretariat, Hobart, 2016. Available from: <https://www.ccamlr.org/en/news/2016/ccamlr-create-worlds-largest-marine-protected-area>.
33. Brooks CM. Competing values on the Antarctic high seas: CCAMLR and the challenge of marine-protected areas. Polar J. 2013; 3: 277-300.
34. Brooks CM, Crowder LB, Curran LM, Dunbar RB, Ainley DG, Dodds KJ, et al. Science-based management in decline in the Southern Ocean. Science 2016; 354: 185-187.
35. ATCM, ATCM XXXVIII – CEP XVIII Information Paper 19. The Scientific Committee on Antarctic Research (SCAR) Annual Report 2014/15. Antarctic Treaty Secretariat, Buenos Aires. 2015. Available from: [www.ats.aq](http://www.ats.aq)
36. CCAMLR. CCAMLR VME Registry. CCAMLR Secretariat, Hobart. 2016. Available from: <https://www.ccamlr.org/en/data/ccamlr-vme-registry>.
37. CCAMLR. Report on bottom fisheries and vulnerable marine ecosystems. CCAMLR Secretariat, Hobart. 2013. Available from: <https://www.ccamlr.org/en/document/publications/2013-report-bottom-fisheries-and-vulnerable-marine-ecosystems>.
38. CCAMLR, CCAMLR Schedule of conservation measures in Force 2015/2016. CCAMLR Secretariat, Hobart. 2016. Available from: <https://www.ccamlr.org/en/document/publications/schedule-conservation-measures-force-2015/16>.
39. Hughes KA, Convey P. The protection of Antarctic terrestrial ecosystems from inter- and intra-continental transfer of non-indigenous species by human activities: a review of current systems and practices. Global Environ Change 2010; 20: 96-112.
40. Hemmings AD, Kriwoken LK. High level Antarctic EIA under the Madrid Protocol: state practice and the effectiveness of the Comprehensive Environmental Evaluation process. Internat Environ Agreem. 2010; 10: 187-208.
41. Dudeney JR, Walton DWH. Leadership in politics and science within the Antarctic Treaty. Polar Res. 2012; 31: 11075.
42. ATCM. Antarctic Treaty Meetings. Antarctic Treaty Secretariat, Buenos Aires. 2012. Available from: <http://www.ats.aq/devAS/ats_meetings.aspx?lang=e>.
43. CCAMLR. CCAMLR Performance Review Panel Report. CCAMLR Secretariat., Hobart. 2008. Available from: <https://www.ccamlr.org/en/system/files/e-Prfrm%20Review%20Report%20Jun09_0.pdf>.
44. Constable AJ, Melbourne-Thomas J, Corney SP, Arrigo KR, Barbraud C, Barnes DK, et al. Climate change and Southern Ocean ecosystems I: how changes in physical habitats directly affect marine biota. Global Change Biol. 2014; 20: 3004-3025.
45. De Broyer C, Koubbi P, Griffiths HJ, Raymond B, d U D'Acoz C, van de Putte A, et al. Biogeographic atlas of the Southern Ocean. Cambridge: Scientific Committee on Antarctic Research; 2014.
46. Stokke OS, Vidas D. Governing the Antarctic. The effectiveness and legitimacy of the Antarctic Treaty System. Cambridge: Cambridge University Press; 1996.
47. Chown SL, Lee JE, Hughes KA, Barnes J, Barrett PJ, Bergstrom DM, et al. Challenges to the future conservation of the Antarctic. Science 2012; 337: 158-159.
48. COMNAP. Antarctic facilities list. COMNAP Secretariat, Christchurch. 2014. Available from: <https://www.comnap.aq/Information/SitePages/Home.aspx>.
49. ATCM. ATCM XXXVII – CEP XVII Working Paper 16. The draft comprehensive environmental evaluation for the construction and operation of the new Chinese research station, Victoria Land, Antarctica. Antarctic Treaty Secretariat, Buenos Aires. 2014. Available from: [www.ats.aq](http://www.ats.aq)
50. ATCM. ATCM XXX – CEP X Information Paper 51. Construction and operation of a new Belgian research station, Dronning Maud Land, Antarctica. Final comprehensive environmental evaluation. Antarctic Treaty Secretariat, Buenos Aires. 2007. Available from: [www.ats.aq](http://www.ats.aq)
51. ATCM. ATCM XXXI – CEP XI Working Paper 5. The draft comprehensive environmental evaluation for the construction and operation of the Chinese Dome. A station in Antarctica. Antarctic Treaty Secretariat, Buenos Aires. 2008. Available from: [www.ats.aq](http://www.ats.aq)
52. ATCM. ATCM XXXIV – CEP XIV Information Paper 64. Final Comprehensive environmental evaluation (CEE) of New Indian research station at Larsemann Hills, Antarctica and update on construction activity. Antarctic Treaty Secretariat, Buenos Aires. 2011. Available from: [www.ats.aq](http://www.ats.aq)
53. ATCM. ATCM XXXVIII – CEP XVIII Information Paper 39. Construction and operation of Belarussian Antarctic research station at Mount Vechernyaya, Enderby Land. Final comprehensive environmental evaluation. Antarctic Treaty Secretariat, Buenos Aires. 2015. Available from: [www.ats.aq](http://www.ats.aq)
54. ATCM. ATCM XXVII – CEP VII Information Paper 94. Are more Antarctic stations justified? Antarctic Treaty Secretariat, Buenos Aires. 2004. Available from: [www.ats.aq](http://www.ats.aq)
55. Molina-Montenegro M, Carrasco-Urra F, Acuña-Rodriguez I, Oses R, Torres-Diaz C, Chwedorzewska K. Assessing the importance of human activities for the establishment of the invasive *Poa annua* in Antarctica. Polar Res. 2014; 33: 21425.
56. Hughes KA, Ireland LC, Convey P, Flemming AH. Assessing the effectiveness of specially protected areas for conservation of Antarctica’s botanical diversity. Conserv Biol. 2016; 30: 113-120.
57. Kennicutt II MC, Chown SL, Cassano JJ, Liggett D, Massom R, Peck LS, et al. Six priorities for Antarctic science. Nature 2014; 512: 23-25.
58. Kennicutt II MC, Chown SL, Cassano JJ, Liggett D, Peck LS, Massom R, et al. A roadmap for Antarctic and Southern Ocean science for the next two decades and beyond. Antarct Sci. 2015; 27: 3-18.
59. Brady A. -M. Editor. The emerging politics of Antarctica. Abingdon: Routledge; 2013.
60. Dodds K, Hemmings AD, Roberts P, editors. Handbook on the politics of Antarctica. London: Edward Elgar; 2017.
61. Österblom H, Folke C. Globalization, marine shifts and the Soviet Union. Phil Trans R Soc B 2015; 370: 20130278.
62. Constable A J. Lessons from CCAMLR on the implementation of the ecosystem approach to managing fisheries. Fish Fisher. 2011; 12: 138-151.
63. Nicol S, Foster J, Kawaguchi S. The fishery for Antarctic krill – recent developments. Fish Fisher. 2012; 13: 30-40.
64. Mallory TG. Fisheries subsidies in China: quantitative and qualitative assessment of policy coherence and effectiveness. Mar Policy 2016; 68: 74-82.
65. IWC. International Whaling Commission Report of the Scientific Committee. Slovenia, Bled. 2016; 7-19 June. Available from: <https://iwc.int/iwc66docs>.
66. Leaper R, Childerhouse S. Present and future conservation management of Antarctic baleen whales. In: Tin T, Liggett D, Maher PT, Lamers M, editors. Antarctic futures. Human engagement with the Antarctic environment. Dordrecht: Springer; 2014. pp. 97-112.
67. Dodds K, Nuttall M. The scramble for the Poles. Cambridge: Polity Press; 2016.
68. Lukin V. Russia’s current Antarctic policy. Polar J. 2014; 4: 199-222.
69. Brooks CM, Ainley DG. Fishing the bottom of the Earth: The political challenges of ecosystem-based management. In: Dodds K, Hemmings AD, Roberts P, editors. Handbook on the politics of Antarctica. London: Edward Elgar; 2017. pp. 893-930.
70. Dodds K, Hemmings KA. Recent developments in relations between the United Kingdom and the Argentine Republic in the South Atlantic/Antarctic region. Polar Rec. 2014; 50: 119-127.
71. Cavanaugh RD, Hill SL, Knowland CA, Grant SM. Stakeholder perspectives on ecosystem-based management of the Antarctic krill fishery. Mar Policy 2016; 68: 205-211.
72. Jacquet J, Pauly D, Ainley DG, Holt S, Dayton P, Jackson J. Seafood stewardship in crisis. Nature 2010; 467: 28-29.
73. Österblom H, Sumaila UR. Toothfish crises, actor diversity and the emergence of compliance mechanisms in the Southern Ocean. Global Environ Change 2011; 21: 972-982.
74. Österblom H, Bodin O. Global cooperation among diverse organizations to reduce illegal fishing in the Southern Ocean. Conserv Biol. 2012; 26: 638-648.
75. Miller DGM. Antarctic marine living resources: The future is not what it used to be. In: Tin T, Liggett D, Maher PT, Lamers M, editors. Antarctic futures. Human engagement with the Antarctic environment. Dordrecht: Springer; 2014. pp. 61-95.
76. Santiago Declaration on the Twenty Fifth Anniversary of the signing of the Protocol on Environmental Protection to the Antarctic Treaty, 30^th^ May 2016. Antarctic Treaty Secretariat, Buenos Aires. 2016. Available from: [www.ats.aq/documents/ATCM39/ad/atcm39_ad003_e.pdf](http://www.ats.aq/documents/ATCM39/ad/atcm39_ad003_e.pdf).
77. IAATO. International Association of Antarctica Tour Operators. IAATO, Newport. 2016. Avalaible from: <http://iaato.org/home>.
78. Tin T, Liggett D, Maher PT, Lamers M, editors. Antarctic futures. Human engagement with the Antarctic environment. Dordrecht: Springer; 2014.
79. Chown SL, Huiskes AH, Gremmen NJM, Lee JE, Terauds A, Crosbie K, et al. Continent-wide risk assessment for the establishment of nonindigenous species in Antarctica. Proc Natl Acad Sci USA 2012; 109:4938-4943.
80. Jacobs S. Observations of change in the Southern Ocean. Phil Trans R Soc A 2006; 364: 1657-1681.
81. Stammerjohn S, Massom R, Rind D, Martinson D. Regions of rapid sea ice change: an inter-hemispheric seasonal comparison. Geophys Res Lett. 2012; 39: L06501.
82. Montes-Hugo M, Doney SC, Ducklow HW, Fraser W, Martinson D, Stammerjohn S, Schofield O. Recent changes in phytoplankton communities associated with rapid regional climate change along the western Antarctic Peninsula. Science 2009; 323:1470-1473.
83. Schloss IR, Abele D, Moreau S, Demers S, Bers AV, Gonzalez O, Ferreyra GA. Response of phytoplankton dynamics to 19-year (1991-2009) climate trends in Potter Cove (Antarctica). J Mar Syst. 2012; 92: 53-66.
84. Atkinson A, Siegel V, Pakhomov EA, Rothery P. Long-term decline in krill stock and increase in salps within the Southern Ocean. Nature 2004; 432: 100-103.
85. Steinberg DK, Ruck KE, Gleiber MR, Garzio LM, Cope JS, Bernard KS, et al. Long-term (1993–2013) changes in macrozooplankton off the Western Antarctic Peninsula. Deep-Sea Res I 2015; 101: 54-70.
86. Turner JN, Barrand N, Bracegirdle T, Convey P, Hodgson DA, Jarvis M. et al. Antarctic climate change and the environment: an update. Polar Rec. 2014; 50: 237-259.
87. Watson RA, Cheung WLW, Anticamara JA, Sumaila UR, Zeller D, Pauly D. Global marine yield halved as fishing intensity redoubles. Fish Fisher. 2013; 14: 493-503.
88. Palomares MLD, Pauly D, editors. Marine fisheries catches of SubAntarctic Islands, 1950 to 2010. Fisheries Research Centre Reports 2015; 23: 1-48.
89. Trivelpiece WZ, Hinke JT, Miller AK, Reiss CS, Trivelpiece SG, Watters GM. Variability in krill biomass links harvesting and climate warming to penguin population changes in Antarctica. Proc Natl Acad Sci USA 2011; 108: 7625-7628.
90. CCAMLR. Report of the XXXIV Meeting of the Commission. CCAMLR Secretariat, Hobart. 2015. Available from: <https://www.ccamlr.org/en/system/files/e-cc-xxxiv_4.pdf>.
91. Abrams PA, Ainley DG, Blight LK, Dayton P, Eastman J, Jacquet J. Necessary elements of precautionary management: implications for the Antarctic toothfish. Fish Fisher. 2016; 17: 1152-1174.
92. Kock K-H. Antarctic fish and fisheries. Cambridge: Cambridge University Press; 1992.
93. Xavier JC, Brandt A, Ropert-Coudert Y, Badhe R, Gutt J, Havermans C, et al. Future challenges in Southern Ocean ecology research. Frontiers Marine Sci. 2016; 3: 94.
94. Shaw JD, Terauds A, Riddle MJ, Possingham HP, Chown SL. Antarctica’s protected areas are inadequate, unrepresentative and at risk. PLoS Biol. 2014; 12: e1001888.
95. Terauds A, Lee J. Antarctic biogeography revisited: updating the Antarctic Conservation Biogeographic Regions. Divers Distrib. 2016; 22, 836-840.
96. Hughes KA, Pertierra LR, Walton DWH. Area protection in Antarctica: how can conservation and scientific research goals be managed compatibly? Environ Sci Policy. 2013; 31: 120-132.
97. Aronson RB, Thatje S, McClintock JB, Hughes KA. Anthropogenic impacts on marine ecosystems in Antarctica. Ann NY Acad Sci 2011; 1223: 82-107.
98. Haward M, Jabour J, Press AJ. Antarctic Treaty System ready for a challenge. Science 2012; 338: 603.
99. Bender NA, Crosbie K, Lynch HJ, Patterns of tourism in the Antarctic Peninsula region: a 20-year analysis. Antarct. Sci. 2016; 28: 194-203.
100. Braun C, Hertel F, Mustafa O, Nordt A, Pfeiffer S, Peter H-U. Environmental assessment and management challenges of the Fildes Peninsula region. In: Tin T, Liggett D, Maher PT, Lamers M, editors. Antarctic futures. Human engagement with the Antarctic environment. Dordrecht: Springer; 2014. pp. 169-191.
101. Tejedo P, Pertierra LR, Benayas J, Convey P, Justel A, Quesada A. Trampling on maritime Antarctica: can soil ecosystems be effectively protected through existing codes of conduct? Polar Res. 2012; 31: 10888.
102. Pertierra LR, Lara F, Tejedo P, Quesada A, Benayas J. Rapid denudation processes in cryptogamic communities from Maritime Antarctica subjected to human trampling. Antarct Sci. 2013; 25: 318-328.
103. Peter H-U, Braun C, Janowski S, Nordt A, Nordt M, Stelter M. The current environmental situation and proposals for the management of the Fildes Peninsula Region. Dessau-Roßlau: Umweltbundesamt; 2013. Available from: <http://www.uba.de/uba-info-medien-e/4424.html>.
104. Tejedo P, Benayas J, Cajiao D, Albertos B, Lara F, Pertierra LR, et al. Assessing environmental conditions of Antarctic footpaths to support management decisions, J Environ Manag. 2016; 177: 320-330.
105. ATCM. Report of the Nineteenth Meeting of the Committee for Environmental Protection (CEP XIX). Antarctic Treaty Secretariat, Buenos Aires. 2016. Available from: [www.ats.aq](http://www.ats.aq).
106. ATCM. Resolution 5 of 2012 – ATCM XXXV – CEP XV. Barrientos Island – Aitcho Islands Visitor Site Guidelines. Antarctic Treaty Secretariat, Buenos Aires. 2012. Available from: [www.ats.aq](http://www.ats.aq).
107. ATCM. Decision 4 of 2010 – ATCM XXXIII – CEP XIII. Liability arising from Environmental Emergencies. Antarctic Treaty Secretariat, Buenos Aires. 2010. Available from: [www.ats.aq](http://www.ats.aq).
108. ATCM. Report of the Committee for Environmental Protection (CEP XVII). Antarctic Treaty Secretariat, Buenos Aires. 2014. Available from: [www.ats.aq](http://www.ats.aq).
109. Lamers M, Haase D, Amelung B. Facing the elements: analysing trends in Antarctic tourism. Tourism Rev. 2008; 63: 15-27.
110. Gutt J. On the direct impact of ice on marine benthic communities, a review. In: Arntz WE, Clarke A, editors. Ecological studies in the Antarctic sea ice zone. Berlin: Springer; 2002. pp. 157-168.
111. Barnes DKA, Conlan C. The dynamic mosaic. In: Rogers AD, Johnston NM, Murphy EJ, Clarke A, editors. Antarctic ecosystems. An extreme environment in a changing world. Oxford: Wiley-Blackwell; 2012. pp. 255-290.
112. Australian Fisheries Management Authority. Heard Island and McDonald Islands Fishery. 2016. Available from: <http://www.afma.gov.au/fisheries/heard-island-mcdonald-island-fishery/>.
113. Australian Government. Heard Island and McDonald Islands Fishery Management Plan 2002, Fisheries Management Plans Amendment 2016. Available from: <https://www.legislation.gov.au/Details/F2016C00640>.
114. Chown SL, Clarke A, Fraser CI, Cary SC, Moon KL, McGeoch MA. The changing form of Antarctic biodiversity. Nature 2015; 522: 431-438.
115. Convey P, Chown SL, Clarke A, Barnes DKA, Bokhorst S, Cummings V, et al. The spatial structure of Antarctic biodiversity. Ecol Monogr. 2014; 84: 203-244.
116. Lee CK, Barbier BA, Bottos EM, McDonald IR, Cary SC. The inter-valley soil comparative survey: the ecology of Dry Valley edaphic microbial communities. Isme J. 2012; 6: 1046-1057.
117. Younger JL, Emmerson LM, Miller KJ. The influence of historical climate change on Southern Ocean marine predator populations: a comparative analysis. Global Change Biol. 2016; 22: 474-493.
118. Constable AJ, de la Mare WK, Agnew DJ, Everson I, Miller DGM. Managing fisheries to conserve the Antarctic marine ecosystem: practical implementation of the Convention on the Conservation of Antarctic Marine Living Resources (CCAMLR). ICES J Mar Sci. 2000; 57: 778–791.
119. McBride MM, Dalpadado P, Drinkwater KF, Godø OR, Hobday AJ, Hollowed AB, et al. Krill, climate, and contrasting future scenarios for Arctic and Antarctic fisheries. ICES J Mar Sci. 2014; 71: 1934-1955.
120. Österblom H, Bodin O, Sumaila UR, Press AJ. Reducing illegal fishing in the Southern Ocean: a global effort. Solutions 2015; 4: 72-79.
121. Croxall JP. The role of science and advocacy in the conservation of Southern Ocean albatrosses at sea. Bird Conserv Int. 2008; 18: s13-s29.
122. Kock K-H, Reid K, Croxall JP, Nicol S. Fisheries in the Southern Ocean: an ecosystem approach. Phil Trans R Soc B 2007; 362: 2333–2349.
123. Ainley AG, Pauly D. Fishing down the food web of the Antarctic continental shelf and slope. Polar Rec. 2014; 50, 92-107.
124. Jacquet J, Blood-Patterson E, Brooks C, Ainley DG. ‘Rational use’ in Antarctic waters. Mar Policy 2016; 63: 28-34.
125. Ainley DG, Nur N, Eastman JT, Ballard G, Parkinson CL, Evans CW, et al. Decadal trends in abundance, size and condition of Antarctic toothfish in McMurdo Sound, Antarctica, 1972-2011. Fish Fisher. 2013; 14: 343-363.
126. Parker SJ, Mormede S, DeVries AL, Hanchet SM, Eisert R. Have Antarctic toothfish returned to McMurdo Sound? Antarct Sci. 2016; 26: 29-34.
127. Welsford DC. Evaluating the impact of multi-year research catch limits on overfished toothfish populations. CCAMLR Sci. 2011; 18: 47-55.
128. Marschoff ER, Barrera-Oro ER, Alescio NS, Ainley DG. Slow recovery of previously depleted demersal fish at the South Shetland Islands, 1983–2010. Fish Res. 2012; 125-126: 206-213.
129. Ashford JR, Croxall JP, Rubilar CS, Moreno CA. Seabird interactions with longlining operations for *Dissostichus eleginoides* at the South Sandwich Islands and South Georgia. CCAMLR Sci. 1994; 1: 143-153.
130. Kock K-H. The direct influence of fishing and fishery-related activities on non-target species in the Southern Ocean with particular emphasis on longline fishing and its impact on albatrosses and petrels – a review. Rev Fish Biol Fisher. 2001; 11: 31-56.
131. Nel DC, Ryan PG, Watkins BP. Seabird mortality in the Patagonian toothfish longline fishery around the Prince Edward Islands, 1996-2000. Antarct Sci. 2002; 14: 151-161.
132. Tuck GN, Polacheck T, Bulman CM. Spatio-temporal trends of longline fishing effort in the Southern Ocean and implications for seabird bycatch. Biol Conserv. 2003; 114: 1–27.
133. Delord K, Gasco N, Weimerskirch H, Barbraud C, Micol T. Seabird mortality in the Patagonan toothfish longline fishery around Crozet and Kerguelen Islands, 2001-2003. CCAMLR Sci. 2005; 12: 53-80.
134. Delord K, Besson D, Barbraud C, Weimerskirch H. Population trends in a community of large Procellariiforms of the Indian Ocean: potential effects of environmental and fisheries interactions. Biol Conserv. 2008; 141: 1840–1856.
135. Tuck GN, Thomson RB, Delord K, Louzao M, Herrera M, Weimerskirch H. An integrated assessment model of seabird population dynamics: can individual heterogeneity in susceptibility to fishing explain abundance trends in Crozet wandering albatross? J Appl Ecol. 2015; 52: 950-959.
136. Ryan PG, Watkins BP. Reducing incidental mortality of seabirds with an underwater longline setting funnel. Biol Conserv. 2002; 104: 127-131.
137. Melvin EF, Sullivan B, Robertson G, Weincke B. A review of the effectiveness of streamer lines as a seabird by-catch mitigation technique in longline fisheries and CCAMLR streamer line requirements. CCAMLR Sci. 2004; 11: 189-201.
138. CCAMLR. Scientific Committee for the Conservation of Antarctic Marine Living Resources. Report of the Thirty-Fourth Meeting of the Scientific Committee. CCAMLR Secretariat, Hobart. 2015. Available from: [www.ccamlr.org](http://www.ccamlr.org).
139. Xiong X, Guardone L, Cornax MJ, Tinacci L, Guidi A, Gianfaldoni D, et al. DNA barcoding reveals substitution of Sablefish (*Anoplopoma fimbria*) with Patagonian and Antarctic Toothfish (*Dissostichus eleginoides* and *Dissostichus mawsoni*) in online market in China: how mislabelling opens door to IUU fishing. Food Control 2016; 70: 380-391.
140. Tuck GN, Phillips RA, Small C, Thomson RB, Klaer NL, Taylor F, et al. An assessment of seabird-fishery interactions in the Atlantic Ocean. ICES J Mar Sci 2011; 68: 1628-1637.
141. Tamini LL, Chavez LN, Góngora ME, Yates O, Rabufetti FL, Sullivan B. Estimating mortality of black-browed albatross (*Thalassarche melanophri*s, Temminck, 1828) and other seabirds in the Argentinean factory trawl fleet and the use of bird-scaring lines as a mitigation measure. Polar Biol. 2015; 38: 1867-1879.
142. Kock KH, Purves MG, Duhamel G. Interactions between cetaceans and fisheries in the Southern Ocean. Polar Biol. 2006; 26: 379-388.
143. Guinet C, Tixier P, Gasco N, Duhamel G. Long-term studies of Crozet Island killer whales are fundamental to understanding the economic and demographic consequences of their depredation behaviour on the Patagonian toothfish fishery. ICES J Mar Sci. 2015; 72: 1587-1597.
144. CCAMLR. Report of the XXVI meeting of the Scientific Committee. CCAMLR Secretariat, Hobart. 2007. Available from: <https://www.ccamlr.org/en/system/files/e-sc-xxvi.pdf>.
145. CCAMLR. Report of the workshop on vulnerable marine ecosystems. CCAMLR Secretariat, Hobart. 2009. Available from: <https://www.ccamlr.org/en/system/files/e-sc-xxviii-a10.pdf>.
146. Martin-Smith K. A risk-management framework for avoiding significant adverse impacts of bottom fishing gear on vulnerable marine ecosystems. CCAMLR Sci. 2009; 16: 177-193.
147. Bargagli R. Antarctic ecosystems. Environmental contamination, climate change and human impact. Berlin: Springer; 2005.
148. Bargagli R. Environmental contamination in Antarctic ecosystems. Sci Total Environ. 2008; 400: 212-226.
149. Corsolini S, Borghesi N, Ademollo N, Focardi S. Chlorinated biphenyls and pesticides in migrating and resident seabirds from East and West Antarctica. Environ Internat. 2011; 37: 1329-1335.
150. Szopińska M, Namieśnik J, Polkwska Ż. How important is research on pollution levels in Antarctica? Historical approach, difficulties and current trends. Rev Environ Contam Toxicol. 2017; 239: 79-156.
151. Ivar do Sul JA, Barnes DKA, Costa MF, Convey P, Costa ES, Campos L. Plastics in the Antarctic environment: are we looking only at the tip of the iceberg? Oecologia Austral. 2011; 15: 150-170.
152. Cipro CVZ, Colabuono FI, Taniguchi S, Montoe RC. Persistent organic pollutants in bird, fish and invertebrate samples from King George Island, Antarctica. Antarct Sci. 2013; 25: 545-552.
153. Bergmann M, Gutow L, Klages M, editors. Marine anthropogenic litter. Heidelberg: Springer; 2015.
154. Gionfrido CM, Tate MT, Wick RR, Schultz MB, Zemla A, Thelen MP, et al. Microbial mercury methylation in Antarctic sea ice. Nature Microbiol. 2016; 1: 16127.
155. Tartu S, Angelier F, Wingfield J, Bustamante P, Labadie P, Budzinski H, et al. Corticosterone, prolactin and egg neglect behavior in relation to mercury and legacy POPs in a long-lived Antarctic bird. Sci Total Environ. 2015; 505: 180-188.
156. Goutte A, Barbraud C, Meillère A, Carravieri A, Bustamante P, Labadie P, et al. Demographic consequences of heavy metals and persistent organic pollutants in a vulnerable long-lived bird, the wandering albatross. Proc R Soc B 2014; 281: 20133313.
157. Wilcox C, Van Sebile E, Hardesty BD. Threat of plastic pollution to seabirds is global, pervasive, and increasing. Proc Natl Acad Sci USA 2015; 112: 11899-11904.
158. Isobe A, Uchiyama-Matsumoto K, Uchida K, Tokai T. Microplastics in the Southern Ocean. Mar Poll Bull. 2017; 114: 623-626.
159. McConnell JR, Maselli OJ, Sigl M, Vallelonga P, Neumann T, Anschütz H, et al. Antarctic-wide array of high-resolution ice core records reveals pervasive lead pollution began in 1889 and persists today. Sci Rep. 2014; 4: 5848.
160. Fuoco R, Capodaglio G, Muscatello B, Radaelloi M. Persistent Organic Pollutants (POPs) in the Antarctic Environment. A Review of Findings. Cambridge: SCAR; 2009. Available from: <http://www.scar.org/scar_media/documents/publications/POPs_in_Antarctica-lowres.pdf>.
161. Douglass AR, Newman PA, Solomon S. The Antarctic ozone hole: an update. Physics Today 2014; 67: 42-48.
162. Kallenborn R, Breivik K, Eckhardt S, Lunder CR, Manø A, Schlabach M, et al. Long-term monitoring of persistent organic pollutants (POPs) at the Norwegian Troll station in Dronning Maud Land, Antarctica. Atmosph Chem Phys. 2013; 13: 6983-6992.
163. Geisz HN, Dickhut RM, Cochran MA, Fraser, Ducklow HW. Melting glaciers: a probable source of DDT to the Antarctic marine system. Environ Sci Technol. 2008; 42: 3958-3962.
164. DeConto RM, Pollard D. Contribution of Antarctica to past and future sea-level rise. Nature 2016; 531: 591-597.
165. Trusel LD, Frey KE, Das SB, Karnauskas KB, Munneke PK, van Meijgaard E, et al. Divergent trajectories of Antarctic surface melt under two twenty-first-century climate scenarios. Nature Geosci. 2015; 8: 927-934.
166. Buelow HN, Kooser AS, Van Horn DJ, Barrett JE, Gooseff MN, Schwartz E, et al. Microbial community responses to increased water and organic matter in the arid soils of the McMurdo Dry Valleys, Antarctica. Frontiers Microbiol. 2016; 7: 1040.
167. Li X, Rignot E, Mouginot J, Scheuchl B. Ice flow dynamics and mass loss of Totten Glacier, East Antarctica from 1989 to 2015. Geophys Res Lett. 2016; 43: 6366-6373.
168. Kennicutt II MC, Klein A, Montagna P, Sweet S, Wade T, Palmer T, et al. Temporal and spatial patterns of anthropogenic disturbance at McMurdo Station, Antarctica. Environ Res Lett. 2010; 5: 034010.
169. Amaro E, Padeiro A, de Ferro AM, Mota AM, Leppe M, Verkulich S, et al. Assessing trace element contamination in Fildes Peninsula (King George Island) and Ardley Island, Antarctic. Mar Pollut Bull. 2015; 97: 523-527.
170. Stark JS, Smith J, King CK, Lindsay M, Stark S, Palmer AS, et al. Physical, chemical, biological and ecotoxicological properties of wastewater discharged from Davis Station, Antarctica. Cold Reg Sci Tech. 2015; 113: 52-62.
171. Wild S, McLagan D, Schlabach M, Bossi R, Hawker D, Cropp R, et al. An Antarctic research station as a source of brominated and perfluorinated persistent organic pollutants to the local environment. Environ Sci Technol. 2015; 49: 103-112.
172. Chen D, Hale RC, La Guardia MJ, Luellen D, Kim S, Geisz HN. Hexabromocyclododecane flame retardant in Antarctica: research stations as sources. Environ Pollut. 2015; 206: 611-618.
173. Emnet P, Gaw S, Northcott G, Storey B, Graham L. Personal care products and steroid hormones in the Antarctic coastal environment associated with two Antarctic research stations, McMurdo Station and Scott Base. Environ Res. 2015; 136: 331-342.
174. Cowan D, Chown SL, Convey P, Tuffin IM, Hughes KA, Pointing S, et al. Non-indigenous microorganisms in the Antarctic: assessing the risks. Trends Microbiol. 2011; 19: 540-548.
175. Hughes KA, Cowan DA, Wilmotte A. Protection of Antarctic microbial communities – ‘out of sight, out of mind’. Frontiers Microbiol. 2015; 6: 151.
176. Ghosh R, Lokman PM, Lamare MD, Metcalf VJ, Burritt DJ, Davison W, et al. Changes in physiological responses of an Antarctic fish, the emerald rock cod (*Trematomus bernacchii*), following exposure to polybrominated diphenyl ethers (PBDEs). Aquat Toxicol. 2013; 128-129: 91-100.
177. Stark JS, Kim SL, Oliver JS. Anthropogenic disturbance and biodiversity of marine benthic communities in Antarctica: a regional comparison. PLoS One 2014; 9: e98802.
178. Corbett PA, King, CK, Stark JS, Mondon JA. Direct evidence of histopathological impacts of wastewater discharge on resident Antarctic fish (*Trematomus bernacchii*) at Davis Station, East Antarctica. Mar Pollut Bull. 2014; 87, 48-56.
179. Corbett PA, King CK. Mondon JA. Application of a quantitative histological health index for Antarctic rock cod (*Trematomus bernacchii*) from Davis Station, East Antarctica. Mar Environ Res. 2015; 109: 28-40.
180. Lister KN, Lamare MD, Burritt DJ. Oxidative damage and antioxidant defence parameters in the Antarctic bivalve *Laternula elliptica* as biomarkers for pollution impacts. Polar Biol. 2015; 38: 1741-1752.
181. Polmear R, Stark JS, Roberts D, McMinn A. The effects of oil pollution on Antarctic benthic diatom communities over 5 years. Mar Pollut Bull. 2015; 90: 33-40.
182. Rodrigues Jr E, Feijó-Oliveira M, Suda CNK, Vani GS, Donatti L, Rodrigues E, et al. Metabolic responses of the Antarctic fishes *Notothenia rossii* and *Notothenia coriiceps* to sewage pollution. Fish Physiol Biochem. 2015; 41: 1205-1220.
183. ATCM. Resolution 3 of 2013 – ATCM XXXVI – CEP XVI. Antarctic Clean-Up Manual. Antarctic Treaty Secretariat, Buenos Aires, 2013. Available from: [www.ats.aq](http://www.ats.aq).
184. Stark JS, Snape I, Riddle MJ, Abandoned Antarctic waste disposal sites: monitoring remediation outcomes and limitations at Casey Station. Ecol Manag Restor. 2006; 7: 21-31.
185. Stark JS, Johnstone GJ, Riddle MJ. A sediment mesocosm experiment to determine if the remediation of a shoreline waste disposal site in Antarctica caused further environmental impacts. Mar Pollut Bull. 2014; 89: 284-295.
186. Barbosa A, de Mas E, Benzal J, Diaz JI, Motas M, Jerez S., et al. Pollution and physiological variability in gentoo penguins at two rookeries with different levels of human visitation. Antarct Sci. 2013; 25: 329-338.
187. Waterhouse E. Ross Sea Region. A State of the Environment Report for the Ross Sea Region of Antarctica. Christchurch: New Zealand Antarctic Institute, Antarctica New Zealand; 2011.
188. Walton DWH, Clarkson PD, Summerhayes CP. Science in the Snow. Fifty Years of International Collaboration through the Scientific Committee on Antarctic Research. Cambridge: Scientific Committee on Antarctic Research; 2011.
189. Hughes KA. Influence of seasonal environmental variables on the distribution of presumptive fecal coliforms around an Antarctic research station. Appl Environ Microbiol. 2003; 69: 4884-4891.
190. Cary SC, McDonald IR, Barrett JE, Cowan DA. On the rocks: the microbiology of Antarctic Dry Valley soils. Nature Rev Microbiol. 2010; 8: 129-138.
191. Camenzuli D, Freidman BL. On-site and in situ remediation technologies applicable to petroleum hydrocarbon contaminated sites in the Antarctic and Arctic. Polar Res. 2015; 34: 24492.
192. Frenot Y, Chown SL, Whinam J, Selkirk PM, Convey P, Skotnicki M, et al. Biological invasions in the Antarctic: extent, impacts and implications. Biol Rev. 2005; 80: 45-72.
193. Hughes KA, Pertierra LR, Molina-Montenegro MA, Convey P. Biological invasions in terrestrial Antarctica: what is the current status and can we respond? Biodiv Conserv. 2015; 24: 1031-1055.
194. Aronson RB, Frederich M, Proce R, Thatje S. Prospects for the return of shell-crushing crabs to Antarctica. J Biogeogr. 2015; 42: 1-7.
195. Kerry KR, Riddle MJ. Health of Antarctic wildlife. A challenge for science and policy. Berlin: Springer; 2009.
196. Grimaldi WW, Seddon PJ, Lyver PO‘B, Nakagawa S, Tompkins DM. Infectious diseases of Antarctic penguins: current status and future threats. Polar Biol. 2015; 38: 591-606.
197. Hughes KA, Pertierra L. Evaluation of non-native species policy development and implementation within the Antarctic Treaty area. Biol Conserv. 2016; 200: 149-159.
198. Hughes KA, Convey P. Determining the native/non-native status of newly discovered terrestrial and freshwater species in Antarctica - Current knowledge, methodology and management action. J Environ Manag. 2012; 93: 52-66.
199. Huiskes AHL, Gremmen NJM, Bergstrom DM, Frenot Y, Hughes KA, Imura S, et al. Aliens in Antarctica: assessing transfer of plant propagules by human visitors to reduce invasion risk. Biol Conserv. 2014; 171: 278-284.
200. Lewis PN, Hewitt CL, Riddle MJ, McMinn A. Marine introductions in the Southern Ocean: an unrecognised hazard to biodiversity. Mar Pollut Bull. 2003; 46: 213-223.
201. Lewis PN, Riddle MJ, Hewitt CL. Management of exogenous threats to Antarctica and the sub-Antarctic Islands: balancing risks from TBT and non-indigenous marine organisms. Mar Pollut Bull. 2004; 49: 999-1005.
202. Lee JE, Chown SL. Temporal development of hull-fouling assemblages associated with an Antarctic supply vessel. Mar Ecol Prog Ser. 2009; 386: 97-105.
203. Lee JE, Chown SL. Quantifying the propagule load associated with the construction of an Antarctic research station. Antarct Sci. 2009; 21: 471-475.
204. Lee JE, Chown SL. Breaching the dispersal barrier to invasion: quantification and management. Ecol Appl. 2009; 19: 1944-1959.
205. Hughes KA, Convey P, Maslen NR, Smith RIL. Accidental transfer of non-native soil organisms into Antarctica on construction vehicles. Biol Invas. 2010; 12: 875-891.
206. Hughes KA, Lee JE, Tsujimoto M, Imura S, Bergstrom DM, Ware C, et al. Food for thought: risks of non-native species transfer to the Antarctic region with fresh produce. Biol Conserv. 2011; 144: 1682-1689.
207. Hughes KA, Ashton GV. Breaking the ice: the introduction of biofouling organisms to Antarctica on vessel hulls. Aquat Conserv*.* 2016. doi: 10.1002/aqc.2625.
208. ATCM. Resolution 3 of 2006 – ATCM XXIX – CEP IX. Ballast Water Exchange. Antarctic Treaty Secretariat, Buenos Aires, 2006. Available from: [www.ats.aq](http://www.ats.aq).
209. CEP. Non-native species manual edition 2011. Antarctic Treaty Secretariat, Buenos Aires, 2011. Available from: [www.ats.aq/documents/atcm34/ww/atcm34_ww004_e.pdf](http://www.ats.aq/documents/atcm34/ww/atcm34_ww004_e.pdf).
210. COMNAP. Non-Native Species Voluntary Checklists. COMNAP Secretariat, Christchurch, 2010. Available from: <https://www.comnap.aq/SitePages/checklists.aspx>.
211. SCAR. Environmental Code of Conduct for Terrestrial Scientific Field Research in Antarctica. SCAR, Cambridge, 2009. Available from: <http://www.scar.org/codes-of-conduct>.
212. Shaw JD. Southern Ocean Islands invaded: conserving biodiversity in the World’s last wilderness. In: Foxcroft LC, Pyšek P, Richardson DM, Genovesi P, editors. Plant invasions in protected areas. Patterns, problems and challenges. Dordrect: Springer; 2014. pp. 449-470.
213. Galera H, Wódkiewicz M, CzyŻ E, Łapiński S, Kowalska ME, Pasik M, et al. First step to eradication of *Poa annua* L. from Point Thomas oasis (King George Island, South Shetlands, Antarctica). Polar Biol. 2016. doi: 10.1007/s00300-016-2006-y.
214. Lee JE, Chown SL. Range expansion and increasing impact of the introduced wasp *Aphidius matricariae* Haliday on sub-Antarctic Marion Island. Biol Invas. 2016; 18: 1235-1246.
215. Gutt J, Bertler N, Bracegirdle TJ, Buschmann A, Comiso J, Hosie G, et al. The Southern Ocean ecosystem under multiple climate change stresses - an integrated circumpolar assessment. Global Change Biol. 2015; 21: 1434-1453.
216. Bednaršek N, Tarling GA, Bakker DCE, Fielding S, Jones EM, Venables HJ, et al. Extensive dissolution of live pteropods in the Southern Ocean. Nature Geosci. 2012; 5: 881-885.
217. Kawaguchi S, Ishida A, King R, Raymond B, Waller N, Constable A, et al. Risk maps for Antarctic krill under projected Southern Ocean acidification. Nature Clim Change 2013; 3: 843-847.
218. Peck VL, Tarling GA, Manno C, Harper EM, Tynan E. Outer organic layer and internal repair mechanism protects pteropod *Limacina helicina* from ocean acidification. Deep-Sea Res. II 2016; 127: 41-52.
219. Bednaršek N, Johnson J, Feely RA.Comment on Peck et al: vulnerability of pteropod (*Limacina helicina*) to ocean acidification: shell dissolution occurs despite an intact organic layer. Deep-Sea Res. II 2016; 127: 53-56.
220. Cross EL, Peck LS, Harper EM. Ocean acidification does not impact shell growth or repair of the Antarctic brachiopod *Liothyrella uva* (Broderip, 1833). J Exp Mar Biol Ecol. 105; 462: 29-35.
221. Peck LS, Morley SA, Richard J, Clark MS. Acclimation and thermal tolerance in Antarctic marine ectotherms. J Exp Biol. 2014; 217: 16-22.
222. Peters GP, Andrew RM, Boden T, Canadell JG, Ciais P, Le Quéré C, et al. The challenge to keep global warming below 2°C. Nature Clim Change 2013; 3: 4-6.
223. Bastmeijer K, van Hengel S. The role of the protected area concept in protecting the world’s largest natural reserve: Antarctica. Utrecht Law Rev. 2009; 5: 7-12.
224. WCPA. Word Commission on Protected Areas. Protected Areas Database. 2014. Available from: [www.protectedplanet.net](http://www.protectedplanet.net).
225. Grant SM, Constable A, Raymond B, Doust S. Bioregionalisation of the Southern Ocean: Report of experts workshop, Hobart, September 2006. WWF-Australia and ACE CRC, Hobart, 2006. Available from: <http://awsassets.wwf.org.au/downloads/mo007_bioregionalisation_of_the_southern_ocean_8sep06.pdf>.
226. Douglass LL, Turner J, Grantham HS, Kaiser S, Constable A, Nicoll R, et al. A hierarchical classification of benthic biodiversity and assessment of protected areas in the Southern Ocean. PLoS One 2014; 9: e100551.
227. Clark GF, Raymond B, Riddle MJ, Stark JS, Johnston EL. Vulnerability of Antarctic shallow invertebrate-dominated systems. Austral Ecol. 2015; 40: 482-491.
228. Rogers AD, Tyler PA, Connelly DP, Copley JT, James R., Larter RD, et al. The discovery of new deep-sea hydrothermal vent communities in the Southern Ocean and implications for biogeography. PLoS Biol. 2012; 10: e1001234.
229. CCAMLR. Conservation Measure 91-03. Protection of South Orkney Islands southern shelf. CCAMLR Secretariat, Hobart, 2009. Available from: <https://www.ccamlr.org/en/measure-91-03-2009>.
230. Welsford DA, Constable A, Nowara GB. The Heard Island and McDonald Islands Marine Reserve and Conservation Zone - A model for Southern Ocean marine reserves? In: Duhamel G, Welsford DA, editors. The Kerguelen Plateau: marine ecosystems and fisheries. Marseille: Société française d'ichtyologie; 2011. pp. 297-304.
231. Lombard AT, Reyers B, Schonegevel LY, Cooper J, Smith-Adao LB, Nel DC, et al. Conserving pattern and process in the Southern Ocean: designing a Marine Protected Area for the Prince Edward Islands. Antarct Sci. 2007; 19: 39-54.
232. Ansorge IJ, Durgadoo JV, Treasure AM. Sentinels to climate change. The need for monitoring at South Africa’s Subantarctic laboratory. S Afr J Sci 2014; 110: Art. #a0044.
233. Collins M, Trathan PN, Grant SM. South Georgia and the South Sandwich Islands Marine Protected Area management plan. Stanley, Falkland Islands: Government of South Georgia and the South Sandwich Islands; 2013.
234. CCAMLR. Scientific Committee for the Conservation of Antarctic Marine Living Resources. Report of the Thirty-First Meeting of the Scientific Committee. CCAMLR Secretariat, Hobart, 2012. Available from: [www.ccamlr.org](http://www.ccamlr.org).
235. ATCM. Summary of the work of the CEP on Marine Protected Areas. Antarctic Treaty Secretariat, Buenos Aires. 2015. Available from: [www.ats.aq/documents/ATCM38/ww/atcm38_ww004_e.pdf](http://www.ats.aq/documents/ATCM38/ww/atcm38_ww004_e.pdf).
236. Jacquet J, Brooks C. Conservation: it is rational to protect Antarctica. Nature 2015; 528: 39.
237. Convey P, Smith RIL, Peat HJ, Pugh PJA, The terrestrial biota of Charcot Island, eastern Bellingshausen Sea, Antarctica: an example of extreme isolation. Antarct Sci. 2000; 12: 406-413.
238. ATCM. Measure 16 of 2013 – ATCM XXXVI – CEP XXXVI. Management Plan for Antarctic Specially Protected Area No. 170 Marion Nunataks, Charcot Island, Antarctic Peninsula. Antarctic Treaty Secretariat, Buenos Aires. 2013. Available from: [www.ats.aq](http://www.ats.aq).
239. Harris CM, Lorenz K, Fishpool LDC, Lascelles B, Cooper J, Coria NR, et al. Important Bird Areas in Antarctica 2015. Cambridge: BirdLife International and Environmental Research & Assessment Ltd.; 2015.
240. Juffe-Bignoli D, Burgess ND, Bingham H, Belle EMS, de Lima MG, Deguignet M, et al. Protected Planet Report 2014. Cambridge: UNEP-WCMC; 2014.
241. UN. The Sustainable Development Goals Report. United Nations, New York, 2016. Available from: <http://unstats.un.org/sdgs/report/2016/The%20Sustainable%20Development%20Goals%20Report%202016.pdf>.
242. ATCM. Resolution 5 of 2015 – ATCM XXXVIII – CEP XVIII. Important Bird Areas in Antarctica. Antarctic Treaty Secretariat, Buenos Aires. 2015. Available from: [www.ats.aq](http://www.ats.aq).
243. Convey P, McInnes SJ. Exceptional tardigrade-dominated ecosystems in Ellsworth Land, Antarctica. Ecology 2005; 86: 519-527.
244. Morgan F, Barker G, Briggs C, Price R, Keys H. Environmental Domains of Antarctica Version 2.0 Final Report. Manaaki Whenua Landcare Research New Zealand Ltd., Wellington. 2007. Available from: <http://www.landcareresearch.co.nz/science/soils-and-landscapes/antarctic-soils/antarctic-environmental-domains>.
245. ATCM. Resolution 3 of 2008 – ATCM XXXI – CEP XI. Environmental Domains Analysis for the Antarctic Continent as a dynamic model for a systematic environmental geographic framework. Antarctic Treaty Secretariat, Buenos Aires. 2008. Available from: [www.ats.aq](http://www.ats.aq).
246. ATCM. Resolution 6 of 2012 – ATCM XXXV – CEP XV. Antarctic Conservation Biogeographic Regions. Antarctic Treaty Secretariat, Buenos Aires. 2012. Available from: [www.ats.aq](http://www.ats.aq).
247. Wilkins D, Yau S, Williams TJ, Allen MA, Brown MV, DeMaere MZ, et al. Key microbial drivers in Antarctic aquatic environments. FEMS Microbiol Rev. 2013; 37: 303-335.
248. Cavicchioli R. Microbial ecology of Antarctic aquatic systems. Nature Rev Microbiol. 2015; 13: 691-706.
249. Holdgate MW. The Antarctic protected areas system in the new Millennium. In: Njaastadt B, editor. Report of the Antarctic Protected Areas Workshop. Trømso: Norsk Polarinstitutt Rapportserie, 1998. Available from: <http://www.ats.aq/documents/cep/first_protected_workshop_e.pdf>.
250. Raymond B. Pelagic Regionalization. In: De Broyer C, Koubbi P, Griffiths HJ, Raymond B, D'Acoz CdU, van de Putte A, et al. editors. Biogeographic atlas of the Southern Ocean. Cambridge: Scientific Committee on Antarctic Research; 2014. pp. 397-403.
251. Lynch MA, Foley CM, Thorne LH, Lynch HJ. Improving the use of biological data in Antarctic management. Antarct Sci. 2016; 28: 425-431.
252. IUCN. World Heritage Outlook. Heard and McDonald Islands. IUCN, Gland. 2014. Available from: <http://www.worldheritageoutlook.iucn.org/search-sites/-/wdpaid/en/145576?p_p_auth=UoU0lsag>.
253. Anonymous. Prince Edward Islands Environmental Management Plan. Department of Environmental Affairs, Pretoria, South Africa. 2016. Available from: <http://www.sanap.ac.za/sanap_ems/docs/marion_ems/PEIMP-Final%20web%20without%20sign/>.
254. CCAMLR. Report of the XXXIII Meeting of the Commission CCAMLR Secretariat, Hobart. 2014. Available from: <https://www.ccamlr.org/en/system/files/e-cc-xxxiii.pdf>.
255. Rogers AD, Yesson C, Gravestock P. A biophysical and economic profile of South Georgia and the South Sandwich Islands as potential large-scale Antarctic protected areas. Adv Mar Biol. 2015; 70: 1-286.
256. Cristofari R, Bertorelle G, Ancel A, Benazzo A, Le Maho Y, Ponganis PJ et al. Full circumpolar migration ensures evolutionary unity in Emperor penguin. Nature Comms. 2016; 7: 11842.
257. Turvey ST. Holocene mammal extinctions. In: Turvey ST, editor. Holocene extinctions. Oxford: Oxford University Press; 2009. pp. 41-62.
258. Tyrberg T. Holocene avian extinctions. In: Turvey ST, editor. Holocene extinctions. Oxford: Oxford University Press; 2009. pp. 63-106.
259. Szabo JK, Khwaja N, Garnett ST, Butchart SHM. Global patterns and drivers of avian extinctions at the species and subspecies level. PLoS One 2012; 7: e47080.
260. Scofield RP. Procellariiform extinctions in the Holocene: threat processes and wider ecosystem-scale implications. In: Turvey ST, editor. Holocene extinctions. Oxford: Oxford University Press; 2009. pp. 151-166.
261. Bonner WN. Conservation in the Antarctic. In: Laws M, editor. Antarctic ecology Volume II. London: Academic Press; 1984. pp. 821-847.
262. Trathan PN, Reid K. Exploitation of the marine ecosystem in the sub-Antarctic: historical impacts and current consequences. Pap Proc R Soc Tasm. 2009;143: 9-14.
263. IUCN. The IUCN Red List of Threatened Species. IUCN, Gland. 2016. Available from: <http://www.iucnredlist.org/>.
264. Phillips RA, Gales R, Baker GB, Double MC, Favero M, Quintana F, et al. The conservation status and priorities for large albatrosses and petrels. Biol Conserv. 2016; 201: 169-183.
265. Trathan PN, García-Borboroglu P, Boersma D, Bost C-A, Crawford RJM, Crossin GT, et al. Pollution, habitat loss, fishing, and climate change as critical threats to penguins. Conserv Biol. 2015; 29: 31-41.
266. Cimino MA, Lynch HJ, Saba VS, Oliver MJ. Projected asymmetric response of Adélie penguins to Antarctic climate change. Sci Rep. 2016; 6: 28785.
267. Springer K. Methodology and challenges of a complex multi-species eradication in the sub-Antarctic and immediate effects of invasive species removal. NZ J Ecol. 2016; 40: 273-278.
268. Lynch HJ, Naveen R, Trathan PN, Fagan W. Spatially integrated assessment reveals widespread changes in penguin populations on the Antarctic Peninsula. Ecology 2012; 93: 1367-1377.
269. Jenouvrier S, Holland M, Stroeve J, Serreze M, Barbraud C, Weimerskirch H et al. Projected continent-wide declines of the emperor penguin under climate change. Nature Clim Change 2014; 4: 715-718.
270. ATCM. Co-Chairs’ Report from Antarctic Treaty Meeting of Experts on Implications of Climate Change for Antarctic Management and Governance. Antarctic Treaty Secretariat, Buenos Aires. 2010. Available from: [www.ats.aq/documents/ATME2010/fr/ATME2010_fr001_e.pdf](http://www.ats.aq/documents/ATME2010/fr/ATME2010_fr001_e.pdf).
271. ATCM. Final Report of the Thirty-ninth Antarctic Treaty Consultative Meeting. Antarctic Treaty Secretariat, Buenos Aires. 2016. Available from: [www.ats.aq](http://www.ats.aq).
272. Clapham PJ, Young SB, Brownell Jr RL. Baleen whales: conservation issues and the status of the most endangered populations. Mammal Rev. 1999; 29: 35-60.
273. Wynen LP, Goldsworthy SD, Guinet C, Bester MN, Boyd IL, Gjertz I, et al. Postsealing genetic variation and population structure of two species of fur seal (*Arctocephalus gazella* and *A. tropicalis*). Mol Ecol. 2000; 9: 299-314.
274. Bonin CA, Goebel ME, Forcada J, Burton RS, Hoffman JI. Unexpected genetic differentiation between recently recolonized populations of a long-lived and highly vagile marine mammal. Ecol Evol. 2013; 3: 3701-3712.
275. Magera AM, Flemming JEM, Kaschner K, Christensen LB, Lotze HK. Recovery trends in marine mammal populations. PLoS One 2013; 8: e77908.
276. Peel D, Bravington M, Kelly N, Double MC. Designing an effective mark-recapture study of Antarctic blue whales. Ecol Appl. 2015; 25: 1003-1015.
277. Tomas PO, Reeves RR, Brownell Jr RL. Status of the world’s baleen whales. Mar Mammal Sci. 2016; 32: 682-734.
278. ATCM. Final Report of the Thirty-fifth Antarctic Treaty Consultative Meeting. Antarctic Treaty Secretariat, Buenos Aires. 2012. Available from: [www.ats.aq](http://www.ats.aq).
279. Jackson JA, Steel DJ, Beerli P, Congdon BC, Olavarría C, Leslie MS, et al. Global diversity and oceanic divergence of humpback whales (*Megaptera novaeangliae*). Proc R Soc B. 2014; 281: 20133222.
280. Torres-Florez JP, Hucke-Gaete R, leDuc R, Lang A, Taylor B, Pimper LE, et al. Blue whale population structure along the eastern South Pacific Ocean: evidence of more than one population. Mol Ecol. 2014; 23: 5998-6010.
281. Grant SM, Hill SL, Trathan PN, Murphy EJ. Ecosystem services of the Southern Ocean: trade-offs in decision-making. Antarct. Sci. 2013; 23: 603-617.
282. Hansen J, Sato M, Hearty P, Ruedy R, Kelley M, Masson-Delmotte V, et al. Ice melt, sea-level rise and superstorms: evidence from paleoclimate data, climate modelling, and modern observations that 2°C global warming could be dangerous. Atmosph Chem Phys. 2106; 16: 3761-3812.
283. Galeotti S, DeConto R, Naish T, Stocchi P, Florindo F, Pagani M, et al. Antarctic ice sheet variability across the Eocene-Oligocene boundary climate transition. Science 2016; 352: 76-80.
284. Landschützer P, Gruber N, Haumann FA, Rödenbeck C, Bakker DCE, van Heuven S, et al. The reinvigoration of the Southern Ocean carbon sink. Science 2015; 349: 1221-1224.
285. McKinley GA, Pilcher DJ, Fay AR, Lindsay K, Long MC, Lovenduski NS. Timescales for detection of trends in the ocean carbon sink. Nature 2016; 530: 469-472.
286. Hodgson-Johnston I, Jackson A, Jabour J, Press A. Cleaning up after human activity in Antarctica: legal obligations and remediation realities. Restor Ecol. 2017; 25: 135-139.
287. McWatters RS, Wilkins D, Spedding T, Hince G, Raymond B, Lagerewskij G, et al. On site remediation of a fuel spill and soil reuse in Antarctica. Sci Total Environ*.* 2016; 571: 963-973.
288. Raymond TC, Snape I. Using triage for environmental remediation in Antarctica. Restor Ecol. 2017; 25: 129-134.
289. Turner J, Bindschadler R, Convey P, di Prisco G, Fahrbach E, Gutt J, et al. Antarctic climate change and the environment. Cambridge: Scientific Committee on Antarctic Research; 2009.
290. ATCM. Resolution 7 of 2005 – ATCM XXVIII – CEP VIII. Biological Prospecting. Antarctic Treaty Secretariat, Buenos Aires. 2005. Available from: [www.ats.aq](http://www.ats.aq).
291. ATCM. ATCM XXXII – CEP XII Working Paper 26. A gap analysis of the Antarctic treaty system regarding the management of biological prospecting. Antarctic Treaty Secretariat, Buenos Aires. 2009. Available from: [www.ats.aq](http://www.ats.aq).
292. ATCM. Resolution 9 of 2009 – ATCM XXXII – CEP XII. Collection and use of Antarctic biological material. Antarctic Treaty Secretariat, Buenos Aires. 2009. Available from: [www.ats.aq](http://www.ats.aq).
293. Lohan D, Johnston S. UNU-IAS Report. Bioprospecting in Antarctica. United Nations University Institute of Advanced Studies, Yokohama. 2005. Available from: <http://collections.unu.edu/eserv/UNU:3100/antarctic_bioprospecting_3.pdf>.
294. Tvedt MW. Patent law and bioprospecting in Antarctica. Polar Rec. 2011; 47: 46-55.
295. Herber BP. Bioprospetcing in Antarctica: the search for a policy regime. Polar Rec. 2006; 42: 139-146.
296. Weber M. Accreditation as a regulatory option for Antarctic bioprospecting. Polar Rec. 2006; 42: 349-357.
297. Hemmings AD. Does bioprospecting risk moral hazard for science in the Antarctic Treaty System? Ethics Sci Environ Polit. 2010; 10: 5-12.
298. Hughes KA, Bridge. Potential impacts of Antarctic bioprospecting and associated commercial activities upon Antarctic science and scientists. Ethics Sci Environ Polit. 2010; 10: 13-18.
299. Joyner CC. Bioprospecting as a challenge to the Antarctic Treaty. In: Hemmings AD, Rothwell DR, Scott KN, editors. Antarctic Security in the Twenty-First Century. Abingdon: Routledge; 2012. pp. 197–214.
300. Puig-Marcó R. Access and benefit sharing of Antarctica's biological material. Mar Genom. 2014; 17: 73-78.
301. ATCM. ATCM XXXIII – CEP XIII Working Paper 2. Biological prospecting in the Antarctic region: a conservative overview of current research. Antarctic Treaty Secretariat, Buenos Aires. 2010. Available from: [www.ats.aq](http://www.ats.aq).
302. ATCM. Resolution 6 of 2013 – ATCM XXXVI – CEP XVI. Biological Prospecting in Antarctica. Antarctic Treaty Secretariat, Buenos Aires. 2013. Available from: [www.ats.aq](http://www.ats.aq).
303. ATCM. Resolution 2 of 2004 – ATCM XXVII – CEP VII. Guidelines for Aircraft near concentrations of birds. Antarctic Treaty Secretariat, Buenos Aires. 2004. Available from: [www.ats.aq](http://www.ats.aq).
304. ATCM. Guidelines for the operation of aircraft near concentrations of birds in Antarctica. Antarctic Treaty Secretariat, Buenos Aires. 2016. Available from: [www.ats.aq/documents/recatt/Att224_e.pdf](http://www.ats.aq/documents/recatt/Att224_e.pdf).
305. ATCM. Resolution 2 of 2011 – ATCM XXXIV – CEP XIV. Revised Guide to the Preparation of Management Plans for Antarctic Specially Protected Areas. Antarctic Treaty Secretariat, Buenos Aires. 2011. Available from: [www.ats.aq](http://www.ats.aq).
306. ATCM. Final Report of the Thirty-eighth Antarctic Treaty Consultative Meeting. Antarctic Treaty Secretariat, Buenos Aires. 2015. Available from: [www.ats.aq](http://www.ats.aq).
307. Scientific Committee on Antarctic Research. SCAR, Cambridge. 2016. Available from: [www.scar.org](http://www.scar.org).
308. ATCM. Resolution 3 of 2015 – ATCM XXXVIII – CEP XVIII. The Antarctic Environments Portal. Antarctic Treaty Secretariat, Buenos Aires. 2015. Available from: [www.ats.aq](http://www.ats.aq).
309. Antarctic Environments Portal. 2016. Available from: [www.environments.aq](http://www.environments.aq).
310. Lynch HJ, Naveen R, Casanovas P. Antarctic Site Inventory breeding bird survey data, 1994-2013. Ecology 2013; 94: 2653.
311. ATCM. ATCM XXXIX – CEP XXXIX Secretariat Paper 5. Five Year Forward Budget Profile. Antarctic Treaty Secretariat, Buenos Aires. 2016. Available from: [www.ats.aq](http://www.ats.aq).
312. UNEP. Biannual Report on the Administration of the Convention on Biological Diversity UNEP/CBD/QR/65. UNEP, Nairobi. 2015. Available from: <http://www.unep.org/>.
313. Njaastad B. Report of the Antarctic Protected Areas Workshop. Norsk Polarinstitutt Rapportserie, Norway. 1998. Available from: <http://www.ats.aq/documents/cep/first_protected_workshop_e.pdf>.
314. Valencia J. Report of the Second Antarctic Protected Areas Workshop. Lima, Peru. Antarctic Treaty Secretariat, Buenos Aires. 1999. Available from: <http://ats.aq/documents/cep/second_protected_workshop_e.pdf2>.
315. De Poorter M. Non-native Species in the Antarctic – Proceedings. Christchurch: Gateway Antarctica Special Publication 0801; 2006.
316. CCAMLR. Report of the CCAMLR Workshop on Marine Protected Areas. Annex 7 to the final report of the 24th meeting of the Scientific Committee. CCAMLR Secretariat, Hobart. 2005. Available from: <https://www.ccamlr.org/en/system/files/e-sc-xxiv.pdf>.
317. ATCM, ATCM XXXII – CEP XII Working Paper 55. Report of the Joint CEP/SC-CAMLR Workshop. 2009. Antarctic Treaty Secretariat, Buenos Aires. 2016. Available from: [www.ats.aq](http://www.ats.aq).
318. ATCM. ATCM XXXIX – CEP XXXIX Information Paper 105. Report on IAATO Operator Use of Antarctic Peninsula Landing Sites and ATCM Visitor Site Guidelines, 2015-16 Season. 2016. Antarctic Treaty Secretariat, Buenos Aires. 2016. Available from: [www.ats.aq](http://www.ats.aq).
319. ATCM. Resolution 4 of 2014 – ATCM XXXVII – CEP XVII. Site Guidelines for visitors. 2014. Antarctic Treaty Secretariat, Buenos Aires. 2016. Available from: [www.ats.aq](http://www.ats.aq).
320. ATCM. ATCM XXXIX – CEP XXXIX Information Paper 121. IAATO Wildlife Watching Guidelines for Emperor Penguins and Leopard Seals. Antarctic Treaty Secretariat, Buenos Aires. 2016. Available from: [www.ats.aq](http://www.ats.aq).
